# Green Corrosion Inhibitors for Metal and Alloys Protection in Contact with Aqueous Saline

**DOI:** 10.3390/ma17163996

**Published:** 2024-08-11

**Authors:** Felipe M. Galleguillos Madrid, Alvaro Soliz, Luis Cáceres, Markus Bergendahl, Susana Leiva-Guajardo, Carlos Portillo, Douglas Olivares, Norman Toro, Victor Jimenez-Arevalo, Maritza Páez

**Affiliations:** 1 Centro de Desarrollo Energético de Antofagasta, Universidad de Antofagasta, Av. Universidad de Antofagasta 02800, Antofagasta 1271155, Chile; markus.bergendahl.fredes@ua.cl (M.B.); susana.leiva.guajardo@ua.cl (S.L.-G.); carlos.portillo@uantof.cl (C.P.); douglas.olivares@uantof.cl (D.O.); 2 Departamento de Ingeniería en Metalurgia, Universidad de Atacama, Av. Copayapu 485, Copiapó 1530000, Chile; 3 Departamento de Ingeniería Química y Procesos de Minerales, Universidad de Antofagasta, Av. Universidad de Antofagasta 02800, Antofagasta 1271155, Chile; luis.caceres@uantof.cl; 4 Facultad de Ingeniería y Arquitectura, Universidad Arturo Prat, Av. Arturo Prat 2120, Iquique 1110939, Chile; notoro@unap.cl; 5 Departamento de Química de los Materiales, Facultad de Química y Biología, Universidad de Santiago de Chile, Av. Libertador B. O’Higgins 3363, Santiago 9170022, Chile; victor.jimenez@usach.cl (V.J.-A.); maritza.paez@usach.cl (M.P.)

**Keywords:** corrosion, green corrosion inhibitors, metals and alloys, saline environment

## Abstract

Corrosion is an inevitable and persistent issue that affects various metallic infrastructures, leading to significant economic losses and safety concerns, particularly in areas near or in contact with saline solutions such as seawater. Green corrosion inhibitors are compounds derived from natural sources that are biodegradable in various environments, offering a promising alternative to their conventional counterparts. Despite their potential, green corrosion inhibitors still face several limitations and challenges when exposed to NaCl environments. This comprehensive review delves into these limitations and associated challenges, shedding light on the progress made in addressing these issues and potential future developments as tools in corrosion management. Explicitly the following aspects are covered: (1) attributes of corrosion inhibitors, (2) general corrosion mechanism, (3) mechanism of corrosion inhibition in NaCl, (4) typical electrochemical and surface characterization techniques, (5) theoretical simulations by Density Functional Theory, and (6) corrosion testing standards and general guidelines for corrosion inhibitor selection. This review is expected to advance the knowledge of green corrosion inhibitors and promote further research and applications.

## 1. Introduction

Corrosion, which is a thermodynamically favored process, is an active and dynamic challenge that affects a wide spectrum of sectors, including (i) industrial equipment and machinery, (ii) the oil and gas industry, (ii) mining and mineral processing, (iv) land and air transportation, (v) infrastructure such as bridges and buildings, (vi) water supply systems, (vii) electrical and electronic devices, (viii) the marine industry, and/or (ix) infrastructure for renewable energy [1,2,3,4,5,6,7,8,9]. This leads to significant economic losses and risks to human safety. Recent technological trends in the field of clean energy and drinking water supply have created the need to handle saline solutions, either at high concentrations or at high temperatures. The exposure of steel to these environments poses a serious condition for corrosion protection in areas involving seawater for desalination, molten salts in solar energy storage [10,11,12,13,14], or saline solutions in copper mining.

Approximately 16,000 operational desalination plants are distributed in 177 countries, which together generate approximately 95 million m^3^/day of freshwater worldwide [15,16,17,18]. In all these cases, which represent aqueous media with a pH close to 7, that is, relatively neutral, corrosion problems have arisen [19,20,21,22]. Corrosion rates in neutral pH environments are reported to undergo significant acceleration, which must be reduced to increase the durability or useful life of the structures. Controlling the corrosion of metals and alloys remains one of the biggest challenges faced by industries in the 21st century. Carbon steels, with their wide applications in various sectors such as chemical and metallurgical industries, construction, agricultural production, medical, and biological processing industries, are chosen mainly for their cost-effectiveness compared to other more resistant alloys like stainless steel. However, the disadvantage of carbon steel is its inability to form protective oxide layers, making the metal surface vulnerable to environmental aggressiveness, particularly when solutions contain Cl^−^ ions. In atmospheric corrosion, there is a competition between reactions of iron with oxygen to form different iron oxides [23,24], with reactions of iron with Cl^−^ ions.

**Figure 1:**
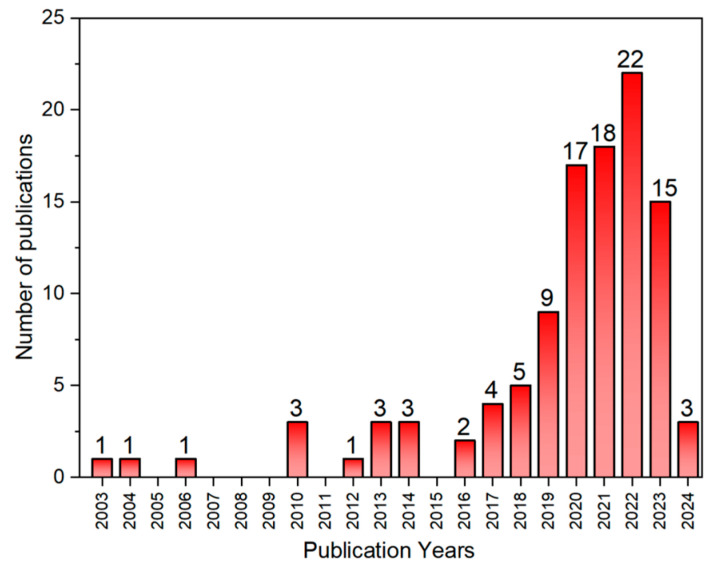
Number of publications per year with the keywords “Green inhibitor corrosion protection neutral” searched in Web of Science.

Regarding corrosion mitigation with inhibitors, the number of articles published on green inhibitors in neutral media, specifically in NaCl solutions, has shown significant growth between the years 2000 and 2023, as shown in Figure 1, for publications that contains specific words of “Green inhibitor corrosion protection neutral” in Web of Science. The growing number of publications reflects a rising interest in the development and research of new corrosion inhibitors that do not alter the environment. These inhibitors are designed to be safe for the atmosphere, soil and water, ensuring they do not harm our planet’s ecosystem. A clear example would be its use to mitigate corrosion in various metal substrates promoted mainly by Cl^−^ ions, such as seawater and freshwater under certain temperature and pH conditions.

The wide range of information generated about corrosion inhibitors under various conditions has been compiled and analyzed in various review manuscripts. Among them, three articles are focused on organic and inorganic inhibitors in ferrous metals in NaCl solutions [25,26,27]. The subjects covered include background information on corrosion, characterization of recently examined materials, inhibition efficiency under laboratory conditions, data interpretation, and general considerations. What is missing is a sequence of steps to validate and test green corrosion inhibitors under industrial conditions.

**Figure 2:**
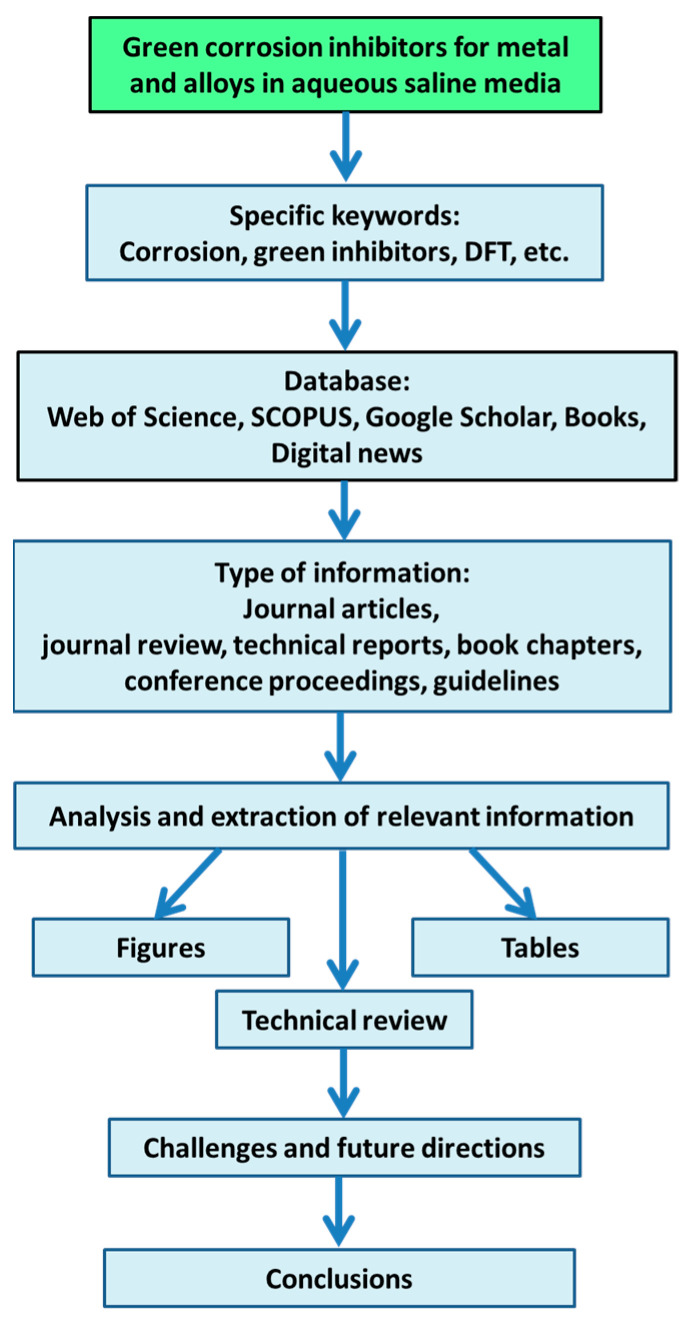
Flow diagram scheme of database collection and methodology for the review and analysis of information on green corrosion inhibitors.

This review aims to provide a comprehensive coverage of the subject, starting from general concepts of corrosion and extending to corrosion testing. It also includes a discussion of practical considerations and guidelines for material selection and testing conditions, which are of paramount importance. We focus on evaluating the efficacy of green inhibitors, specifically those used in NaCl solutions, with special emphasis on plant extracts as the main source of environmentally friendly corrosion inhibitors. Explicitly, the following aspects are covered: (1) attributes of corrosion inhibitors, (2) general corrosion mechanism, (3) mechanism of corrosion inhibition in NaCl, (4) typical electrochemical and surface characterization techniques, (5) theoretical simulations by Density Functional Theory, and (6) corrosion testing standards and general guidelines for corrosion inhibitor selection. To achieve this, a literature review was conducted across scientific database such as Web of Science, SCOPUS, Google Scholar, and digital news, complemented by books related to Chemical Engineering, Electrochemistry, and Corrosion Science. Specific keywords used in the search included green inhibitors, plant extracts, NaCl, saline solution, corrosion, DFT, inhibition efficiency, and others. The literature information was filtered and refined to focus on plant-based corrosion inhibitors for metallic metals and alloys exposed to aggressive saline environments. Figure 2 presents a scheme that summarizes the general methodology of literature collection and analysis. Overall, this paper is expected to advance the knowledge of green corrosion inhibitors and promote further research and its applications.

## 2. General Corrosion Mechanism and the Influence of the Chloride Ion

Corrosion, defined as a natural and spontaneous process, causes substantial economic losses globally. According to the US National Association of Corrosion Engineers (NACE), the cost of corrosion amounts to approximately USD 2.5 trillion per year, comparable to approximately 3.4% of the gross domestic product (GDP) [28,29,30]. However, it is estimated that the implementation of an effective corrosion prevention and control method would reduce the associated annual costs by 15–35% [31]. Specific examples of severe infrastructure impacts include catastrophic events, such as bridge collapses, drilling string failures, and severe damage to chemical plants [32,33,34,35,36]. 

Many illustrative incidents have occurred in the past. To name a few: (i) in 2002, Prestige, an embarked oil tanker, sailed with a major crack in its hull, causing nearly 15,000 tons of fuel to spill into the sea, culminating in the rupture of the ship and releasing around 77,000 tons of fuel oil into the sea [37]; and (ii) in 2018, the Morandi Bridge in Genoa, Italy, collapsed because of corrosion problems [38,39]. A practical and cost-effective alternative to avoid corrosion is the use of corrosion inhibitors, which are now required by environmental regulations to be green or environmentally friendly. In general, a green inhibitor is a substance derived from natural products that have corrosion-inhibiting properties and are environmentally friendly [40]. The following sections provide an explanation that is more detailed.

In neutral media such as seawater, in the presence of molecularly dissolved oxygen, the cathodic reactions are the oxygen reduction reaction (ORR) and the hydrogen evolution reaction (HER). The direct route during the ORR process consumes 4e^−^ during the reaction and is considered the most energy efficient, and is expressed as follows in Equation (1) [41]:

(1) $$O_{2}+2H_{2}O+4e^{-}\to 4OH^{-}$$

The indirect route considers a sequential consumption of (2 + 2)e^−^ and generates hydroperoxide (HO_2_^−^) as an intermediate product during the ORR process. This reaction reduces the energy efficiency of the system and is expressed as follows [42]:

(2) $$O_{2}+H_{2}O+2e^{-}\to H{O_{2}}^{-}+{OH}^{-}$$

(3) $$H{O_{2}}^{-}+H_{2}O+2e^{-}\to {3OH}^{-}$$

The decomposition of HO_2_^−^ takes place via a catalytic reaction as expressed in Equation (4):

(4) $$H{O_{2}}^{-}\to \frac{1}{2}O_{2}+{OH}^{-}$$

The HO_2_^−^ formation on the metallic electrode surface depends upon the surface state [43,44].

The HER mechanism plays an essential role in electrochemical devices, such as electrolyzer devices for green H_2_ production. The HER exhibits cathodic window potentials (−1000 mV/ SHE; Standard Hydrogen Electrode). In saline solutions, metals facilitate the HER by reducing hydrogen protons (H^+^) adsorbed from water molecules, promoting the crevice corrosion process and producing the evolution of H_2_ gas from the metal surface, as expressed in Equation (5):

(5) $${2H}^{+}+{2e}^{-}\to H_{2}$$

By controlling the anodic and/or cathodic reactions through the implementation of corrosion inhibitors, protective coatings, or anodic/cathodic protection techniques, the adverse impact of corrosion can be mitigated, preserving the structural integrity of the metal components in contact with neutral environments [45].

In NaCl electrolytes, two primary forms of corrosion are typically involved in the metallic degradation. The first is crevice corrosion, which is associated with the cathodic subprocess and occurs within confined spaces or crevices, where the dissolved oxygen concentration is typically lower and H^+^ ions are adsorbed. The second form is pitting corrosion, which occurs during the anodic subprocess when the metal is dissolved due to contact with aggressive ions, such as Cl^−^ ions, present in the electrolyte. The excess of cationic ions released during the corrosion process generates electrostatic compensating by the migration of anions ions, such as Cl^−^ and OH^−^, into the crevice from the saline electrolyte bulk. Consequently, O_2_ within the crevice becomes depleted, but the ORR persists at another site on the metallic surface. This ion accumulation amplifies the corrosive environment, exacerbating localized corrosion effects according to the following mechanisms:

(6) $$M^{n+}+{nCl}^{-}\to {MCl}_{n}$$

(7) $${MCl}_{n}+{nH}_{2}O\to {M(OH)}_{2}+{nH}^{+}+{nCl}^{-}$$

(8) $$M^{n+}+{nH}_{2}O\to {M(OH)}_{2}+{nH}^{+}$$

where M represents the metal and M^n+^ represents the metal ions of valence n.

The electrolyte around the crevice contains Cl^−^ and H^+^ ions, which attack and deteriorate the protective passive film, transitioning from a passive to an active state. Consequently, this transformation significantly accelerates metal dissolution driven by autocatalytic reactions [46]. 

Anodic dissolution or pitting corrosion affects the metal (M). The Cl^−^ ions can preferentially adsorb on the metal surface competing with other species. The anodic reaction is expressed by Equation (9):

(9) $$M\to M^{n+}+{ne}^{-}$$

The adsorbed Cl^−^ ions will break down the passive film formed on the metal surface and thus increase the corrosion rate. In the absence of Cl^−^ ions in the electrolyte, the rate of anodic dissolution is kinetically controlled by a charge-transfer step, and the rate-determining process is described as follows:

(10) $${M\cdot H}_{2}O_{ads}\to {M\cdot OH}_{ads}+H^{+}+e^{-}$$

(11) $${{M\cdot OH}_{ads}}^{-}\to {M\cdot OH}_{ads}+e^{-}$$

Furthermore, when the Cl^−^ ion is present in the electrolyte, the OH^−^ ions must compete with Cl^−^ for the surface sites. Therefore, reactions via a Cl^−^ pathway occur in parallel with reactions via an H_2_O pathway, following the reactions:

(12) $${M\cdot H}_{2}O_{ads}+{Cl}^{-}\to {M\cdot Cl}_{ads}+H_{2}O+e^{-}$$

(13) $${{M\cdot OH}_{ads}}^{-}+{Cl}^{-}\to {M\cdot Cl}_{ads}+{OH}^{-}+e^{-}$$

At a constant electrode potential, the reaction rate for the Cl^−^ the pathway is faster than the water pathway for all pH values, thus increasing the corrosion rate. The corrosion acceleration effect due to the Cl^−^ ions on the anodic dissolution are realized by forming an intermediate connecting structure that is usually described as the “catalytic mechanism” [47]. However, it is important to note that the behavior of different types of corrosion in NaCl environments can be complex and influenced by multiple factors. Adequate corrosion protection strategies, such as the use of inhibitors or selection of corrosion-resistant materials, are crucial for mitigating the effects of corrosion in NaCl environments and ensuring the longevity and integrity of metal structures. Early studies have identified several ways in which Cl^−^ ions influence corrosion, including catalytic processes (adsorption theory), effects on oxide films, and dissolved gas effects. Table 1 provides bibliographic details regarding the materials and plant-based compounds used as eco-friendly corrosion inhibitors when exposed to electrolytes such as seawater, saline, and hypersaline solutions. This shows a diverse range of green inhibitors suitable for different environments and applications, highlighting their versatility in industrial settings.

In a NaCl environment, corrosion generally occurs through electrochemical reactions involving the dissolution/oxidation of the metal (anodic half-reaction) and the reduction of O_2_ (cathodic half-reaction) and/or, depending on the pH of the medium, the reduction of hydrogen ions (cathodic half-reaction), which entails the evolution of hydrogen, H_2_. The anodic reaction releases metal cations and electrons; the metal ions, depending on the pH of the medium, remain as ions (acidic pH) or quickly combine with oxygen ions, generating the corresponding metal oxides/hydroxides. The cathodic reaction, for its part, consumes these electrons, generating H_2_O/OH^−^ in the case that the cathodic reaction is the reduction of oxygen, or generating hydrogen in the case that the cathodic reaction corresponds to the reduction of hydrogen ions, H^+^. In any case, charge transfer reactions, oxidation, and reduction contribute to the overall corrosion process. As a result, metal surfaces degrade, causing material loss and structural deterioration.

As can be observed from the above discussion, numerous environmental and material factors influence the rate and severity of corrosion in neutral aqueous environments in the presence of NaCl. Temperature, humidity, Cl^−^ ion concentration, and pH levels play crucial roles in determining the corrosion potential of a given metallic system. Additionally, factors associated with the material in question, such as composition, microstructure, and metallurgical history, also affect the corrosion susceptibility. Metals with higher reactivity and weaker passive layers tend to exhibit greater vulnerability to corrosion in neutral NaCl environments. Furthermore, the surface regions associated with the presence of impurities, second phases, and grain boundaries represent sites of high surface energy, where the probability of generating localized corrosion is greater, exacerbating the general degradation process [46,47,48,49,50,51]. During the corrosion process, metal ions combine with Cl^−^ ions to form various corrosion products, including metal chlorides and oxides. Although certain corrosion products can provide a barrier capable of retarding further corrosion, the morphology of the corrosion product is decisive, giving relative passivity to the metal surface. If the morphology is porous, the entrance of aggressive species into the metal-environment interface and the occurrence of electrochemical reactions would lead to more extensive degradation of the material [52,53].

## 3. Attributes of Green Corrosion Inhibitors

Green inhibitors have been progressively integrated into corrosion management methodologies that perform comprehensive life cycle assessments (LCAs) to assess their aggressive environmental impacts throughout their life cycle [54,55,56,57]. Integrating green inhibitors into corrosion management strategies requires ensuring their long-term performance and durability. Corrosion protection is often a long-term requirement, particularly for infrastructure and industrial applications. Therefore, green inhibitors must demonstrate long-lasting inhibitory efficacy and resist degradation or depletion over long periods. Long-term studies and field trials are essential to validate the sustained performance of green inhibitors and instilling confidence among end users and stakeholders. This work presents a focused review of the efficacy of green inhibitors, with special emphasis on plant extracts as a primary and environmentally friendly source for corrosion protection.

Plant-based green inhibitors for corrosion protection have demonstrated notable inhibitory properties, establishing their efficacy as valuable candidates for corrosion-management strategies [58,59,60]. These green inhibitors act through a variety of mechanisms, such as (i) the formation of a protective film on the metal surface, (ii) inhibition of electrochemical reactions that cause corrosion, or (iii) substantive reduction in the transfer of corrosive substances from the environment to the metal surface. The performance of many green inhibitors has been comparable or superior to that of their traditional counterparts in laboratory and field tests, but their action time is shorter than that of commercial corrosion inhibitors. Integrating green inhibitors into corrosion protection strategies not only benefits robust corrosion resistance but also addresses the growing demand for environmentally friendly practices that align with the principles of sustainability and responsible management of resources. Therefore, it is vital to evaluate the compatibility of green inhibitors with various coating materials, surface preparation techniques, and application methods to ensure a seamless transition. Furthermore, research efforts should focus on optimizing the combination of green inhibitors with other corrosion protection strategies, such as cathodic protection, sacrificial anode systems, and coatings, to improve the overall effectiveness of the corrosion management approach [61].

Most of the current commercial corrosion inhibitors are generally composed of chromates (CrO_4_^2−^), phosphates (PO_4_^3−^), nitrates (NO_3_^−^), and borates (BO_3_^3−^). Unfortunately, these substances exhibit considerable toxicity or possess compositions with significant environmental impact. Therefore, there is an urgent need to explore potential alternatives for corrosion inhibitors that not only offer robust protection against the corrosion process or that surpass the effectiveness of inhibitors currently used, but also that their use does not cause harm to the environment and/or to the living organisms present in that environment. Compared to commercial corrosion inhibitors, eco-friendly corrosion inhibitors show several individual characteristics as sustainable and environmentally friendly alternatives. Some of these features are (i) biodegradability: green corrosion inhibitors are derived from biodegradable and renewable resources, meaning that natural processes can break them down, minimizing their impact on the environment; (ii) low toxicity: green corrosion inhibitors are non-toxic to humans, animals, and aquatic life; (iii) efficacy: green corrosion inhibitors show significant inhibitory properties that prevent or slow down the corrosion process or mechanisms; (iv) renewable supply: green corrosion inhibitors are materials from plant extracts, natural polymers, or biopolymers, which reduce dependence on non-renewable resources and promote sustainability; and (v) low environmental impact: green corrosion inhibitors are created to minimize the impact on ecosystems and natural environments, reducing the carbon footprint through the implementation of sustainable practices. Natural extracts derived from a variety of plant species have shown promising corrosion-inhibition properties in a wide range of aqueous and chlorinated environments. These plant extract-based inhibitors have gained popularity as effective alternatives in various aqueous environments, demonstrating their potential to address corrosion protection challenges in an environmentally sustainable manner [62,63].

## 4. Mechanism of Corrosion Inhibition in NaCl

**Figure 3:**
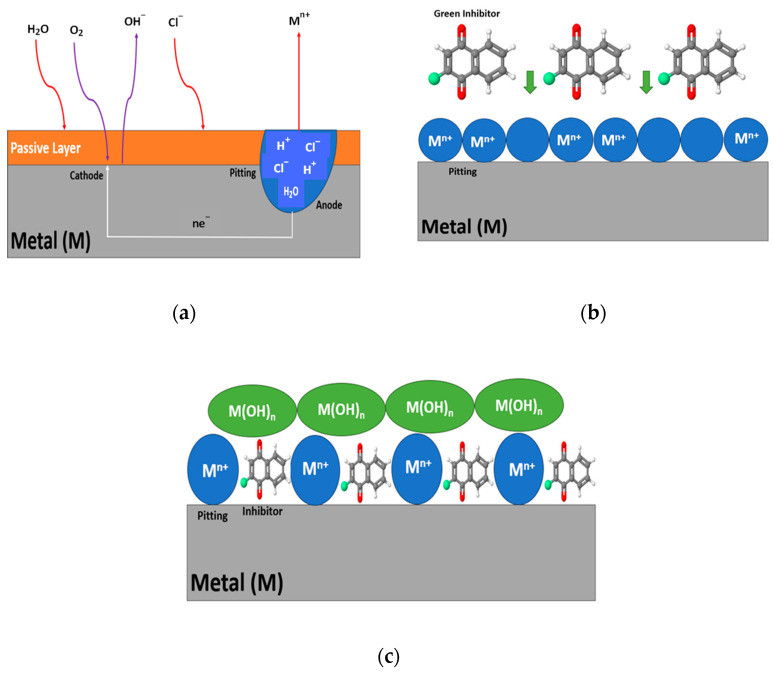
(**a**) Cathodic–anodic processes due to Cl^−^ ions without a green inhibitor, (**b**) passivating mechanism of anodic green inhibitor, and (**c**) organic compounds acting as a film through surface adsorption, forming cathodic or anodic products known as rust. Based on Kumar et al.’s results [64], where O-atoms of the *Lawsonia inermis* molecule form covalent bonds with Fe atoms of the metallic surface (red ball: oxygen, white ball: hydrogen, gray ball: carbon, and green ball: hydroxide).

Green corrosion inhibitors aim to reduce the electrochemical mechanisms on the metal surface when they are in direct contact with aggressive environments. Essentially, any chemicals capable of slowing down the corrosion rate qualify as inhibitors [32]. These inhibitors are typically chemical compounds available in liquid, gas, or combined forms. The inhibitor functions through two fundamental processes: first, transportation of the active component to the metal surface, followed by its interaction with the metallic surface. Corrosion inhibitors often consist of functional groups containing N, S, O, Ar, and different heavy metals such as Zn, Pb, Bi, Cd, and Hg in their composition. Furthermore, organic inhibitors used for corrosion protection can be operated as cathodic, anodic, or mixed (cathodic–anodic) inhibitors by forming a protective film over the surface via complex adsorption mechanisms (see Figure 3).

Green corrosion inhibitors are generally composed of an aqueous emulsion of esters and amino alcohols, which were initially believed to function solely as cathodic inhibitors. However, green corrosion inhibitors exhibit reactivity at both cathodic and anodic sites, effectively reducing the corrosion rate. In the adsorption mechanism, green corrosion inhibitors form a thin hydrophobic protective layer that covers the entire surface of the metal. The formation of this layer is promoted by polar hydrophobic groups, such as N, S and OH^−^, which contribute to the protection efficiency, leading to these inhibitors often being referred to as adsorption or film formation inhibitors. The effectiveness of a film is influenced by the chemical and molecular functions of the inhibitor and the affinity of the metal surface. Commonly, inhibitors, amino alcohols, and amino acids are combined for their ability to dislocate Cl^−^ ions and form a durable passive layer. The versatility and protective qualities of these organic inhibitors make them valuable assets in corrosion protection strategies, offering improved performance and durability for metallic structures.

**Figure 4:**
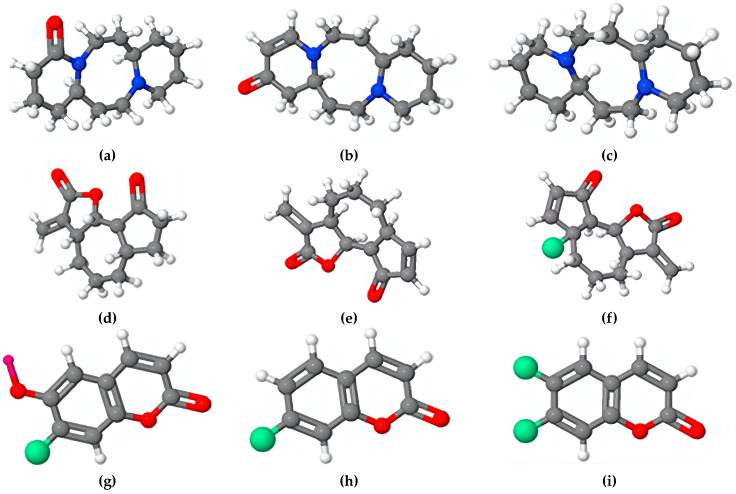
Molecules structures derived from Lupine extracts. (**a**) lupanine, (**b**) multiflorine, (**c**) sparteine and damsissa extract (**d**) damsin, (**e**) ambrosin, (**f**) parthenin, (**g**) scopoletin, (**h**) umbelliferone, and (**i**) esculetin. Red ball: oxygen, white ball: hydrogen, gray ball: carbon, green ball: hydroxide, pink ball: CH_3_, and blue ball: nitrogen.

Figure 4 illustrates the primary structures of the organic compounds used as green corrosion inhibitors, which act by forming a protective film on the metal surface through an adsorption mechanism. For example, Abdel-Gaber et al. [65] have studied the effect of lupanine and damsissa extracts on the corrosion of steel in sodium sulfate with and without NaCl. The authors report the chemical formulas of some constituents of lupine extract, such as lupanine and multiflorine, and constituents of damsissa extract, such as sparteine, damsin, ambrosin, parthenin, scopoletin, umbelliferone, and esculetin (Figure 4), which act as anodic type inhibitors.

**Figure 5:**
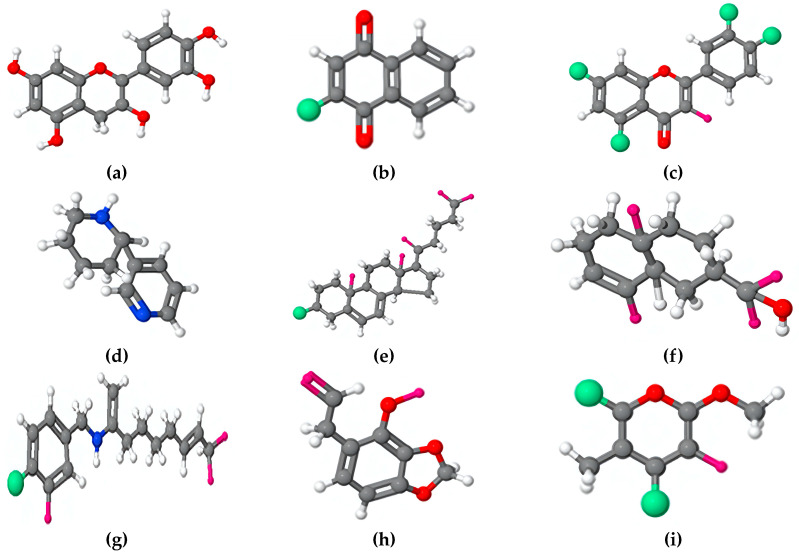
Molecules structures of active compounds present in plant-based corrosion inhibitors. (**a**) catechin, (**b**) *Lawsonia inermis*, (**c**) *Hibiscus rosa-sinensis Linn* (Quercetin-3-O-glucoside), (**d**) anabasine, (**e**) 7-dehydrocholesterol, (**f**) halfabar, (**g**) capsaicin, (**h**) croweacin, (**i**) chitosan. Red ball: oxygen, white ball: hydrogen, gray ball: carbon, and green ball: hydroxide, pink ball: CH_3_, blue ball: nitrogen.

The film formed on the metallic surface serves as a cohesive, impenetrable coating that protects the metal from an aggressive environment and obstructs the penetration of corrosive agents, aggressive ions, and moisture, thereby reducing the rate of corrosion. Figure 5 shows organic structures of different compounds present in plant extracts such as cachetin [66,67,68], *Lawsonia inermis* [69,70], *Hibiscus rosa-sinensis Linn* (Quercetin-3-O-glucoside) [71], nicotiana [72], halfabar [73], capsaicin [74], croweacin [75], and chitosan [76], which can be act as corrosion inhibitor by formation of an protective film on the metallic surface limiting the corrosion. The driving forces that entail the formation of a film of the organic compounds, together with its hydrophobic property, are crucial characteristics to avoid direct contact between the metal and the corrosive environment, thus effectively mitigating the corrosion process and extending the useful life of the protected material.

The functional groups that are part of the molecular structure of corrosion inhibitor compounds play an important role on the surfaces of active-passive metals. Chemical affinities and high local surface energies, which are associated with the presence of defects, intermetallic particles, or simple morphological irregularities, synergistically facilitate the adsorption of inorganic, organic, and hybrid compounds. The adsorption intensity is related to the durability of the surfaces protected by these inhibitors. Thus, different chemical and physical surface treatments have been suggested to modulate the adsorption energy.

**Figure 6:**
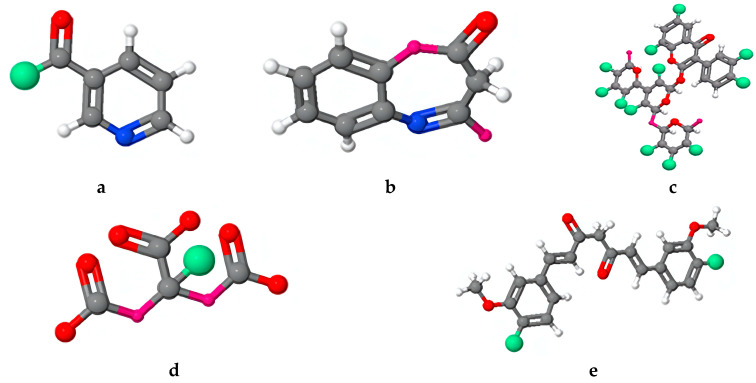
Molecules structures of active compounds present in plant-based corrosion inhibitors: (**a**) niacin, (**b**) benzodiazepine, (**c**) *Catharanthus roseus*, (**d**) citrate, (**e**) curcumin. Red ball: oxygen, white ball: hydrogen, gray ball: carbon, and green ball: hydroxide, pink ball: CH_3_, blue ball: nitrogen.

Figure 6 shows molecules of organic compounds typically used as corrosion inhibitors for steel. Studies have shown that the adsorption energies of vitamin B3 (niacin) on steel were in the range of −1.30 to −2.61 eV, and for the vitamin B6 and vitamin C were −3.87 and −3.88 eV, respectively, indicating that these compounds occupy the reactive O sites of the steel surface, preventing the dissociation of water molecules [77]. Benzodiazepine molecules, which is present in different medical plants and foods, have revealed a ΔG_ads_ higher than −40 kJ mol^−1^, indicating that the adsorption mechanism between MHBZO molecules and copper surface is likely a chemisorption [78]. A similar adsorption mechanism thought chemisorption was founded for extracts of Catharanthus roseus on mild steel in NaCl [79], in contrast to citrate molecules, which are electrostatically adsorbed on the anion-covered surface [80]. Furthermore, curcumin have revealed an adsorption capacity on metallic surface in acid, neutral, and saline mediums through a Langmuir isotherm mechanism [81].

Theoretical or computational chemistry has played an important role in understanding the variables that dominate the intensity of adsorption, which has subsequently been demonstrated experimentally. The concept of modulating the surfaces for specific purposes is fascinating. For example, metallic surfaces are modulated for electrocatalysis by the adsorption of compounds containing active sites, which are generally metallic centers that facilitate the charge transfer processes that occur on the modified surface and are associated with an electrochemical reaction of interest, such as electroreduction for oxygen, which is associated with the development of fuel cells.

On the other hand, in the corrosion and protection of metal surfaces, the aim is to inhibit charge transfer processes, thus hindering the local and/or general degradation of the surface. The differences in the chemical lability of the different metals, as well as the molecular configuration of the inhibitor compounds, and more importantly, the complex and variable composition of the medium means that the inhibitory effect does not always persist over time. This represents a great challenge for the design and development of new corrosion inhibitors, such as those associated with so-called green inhibitors, because they must not only limit the charge transfer processes but also provide high durability to the metal surface. Ideally, they should be degraded in an environmentally friendly manner.

**Table 1:** Overview of environmentally friendly green corrosion inhibitors.

Item	Scientific Name	Common Name	Plant Material	Family	Extraction Solvent	Metal Specimen	Electrolyte	Temp °C	Adsorption Isotherm	IE, %	Type of Inhibition	Scan Rate (mV/s)	Time in Contact
[66]	*Rosmarinus officinalis* L.	Romero/Rosemary	Leaves	Lamiaceae	Water	Al-Mg	3% NaCl	25	Freundlich	93.8	Cathodic	2	30 min
[65]	*Ambrosia maritima*	Damsissa	Dried	Asteraceae	Water	Steel	0.5 M Na_2_SO_4_ (NaCl 0.01 to 0.1 M)	30	---	~65	Anodic	0.3	20 min
[65]	*Lupinus*	Lupine	Dried	Fabaceae	Water	Steel	0.5 M Na_2_SO_4_ (NaCl 0.01 to 0.1 M)	30	---	~68	Anodic	0.35	20 min
[73]	*Lupinus*	Lupine	Seeds	Fabaceae	Water	Zn	0.5 M NaCl	30	Langmuir, and Florry-Huggins	89.1	Mixed	0.3	---
[73]	*Cymbopogon proximus*	Halfabar	Seeds	Poaceae	Water	Zn	0.5 M NaCl	30	Langmuir, and Florry-Huggins	94.7	Mixed	0.3	---
[73]	*Ambrosia maritima*	Damsissa	Seeds	Asteraceae	Water	Zn	0.5 M NaCl	30	Langmuir, and Florry-Huggins	90.7	Mixed	0.3	---
[75]	*Ammi visnaga* L.	Khella	Leaves and flowers	Apiaceae	Hydrodistillation	Cu-Zn	3% NaCl	25	---	93.04	Mixed	0.5	60 min
[78]	4-methyl-1,2-dihydro-3H-benzo[b][1,4]diazepin-3-one (MHBZO)	benzodiazepine	---	---	Diethylether	Cu	3.5% NaCl	25	Langmuir	96	Mixed	1	24 h
[79]	*Catharanthus roseus*	Vinca rosea	Root and stem	Apocynaceae	Etanol	Mild steel	3.5% NaCl	35	---	70	Mixed	0.5	5 days
[82]	Honey	Natural honey	---	---	---	Carbon steel A106	Saline water	---	Langmuir	91.23	Anodic	---	5 days
[83]	Tobacco Varierity NC129, KY171, MD609, B005 and Little Crittenden	Tobacco	Cured leaves, dried leaves, stems and twigs, tobacco dust	Solanaceae	Water	1008/1010 cold-rolled steel 3105 H24/Al Q-panels	1% NaCl	20	---	---	Anodic	---	25 days
[84]	*Tinospora cordifolia*	Guduchi	Stem	Menispermaceae	Methanol	Carbon steel	Seawater	27, 37, 47	Langmuir	85.94	Mixed	1	0, 30 and 60 min
[85]	*Ricunus communis*	Castor bean	Leaves	Euphorbiaceae	Ethanol	Mild steel	100 ppm NaCl	20	---	84	Mixed	1	4 days
[86]	*Ricinus communis*	Castor bean	Fruits	Euphorbiaceae	Methanol	Steel	3.5% NaCl	---	Temkin	79	Mixed	---	120 days
[87]	*Lawsonia inermis*	Henna tree	Leaves	Lythraceae	Water	Carbon steel L-52	0.1 M HCl, 3.5% NaCl and 0.1 M NaOH	30	Langmuir	95.78	Mixed	---	---
[88]	*Hibiscus rosa-sinensis* Linn	China rose/Rose mallow	Petals	Malvaceae	Water	Carbon steel	60 ppm Cl^−^ (Absence and presence of Zn^2+^)	---	---	98	Mixed	10	1 day
[89]	*Ambrosia maritima*, L.	Damsissa	Dried	Asteráceas	Water	Al	2 M NaOH in 0.5 M NaCl	25, 30, 35, 40	---	88.3	Mixed	0.3	35 days
[90]	*Camellia sinensis*	Green Tea	Dried	Theaceae	Water	AISI 304, Carbon steel and brass sheets	3.5% NaCl	20, 50	---	---	---	0.25	30–60–90 days
[90]	*Nicotiana tabacum*	Tobacco	Dried	Solanaceae	Water	AISI 304, Carbon steel and brass sheets	3.5% NaCl	20, 50	---	---	---	0.25	30–60–90 days
[90]	*Rizophora*	Rizophora	Dried	Rhizophoraceae	Water	AISI 304, Carbon steel and brass sheets	3.5% NaCl	20, 50	---	---	---	0.25	30–60–90 days
[90]	*Mimosa púdica*	Mimosa	Dried	Fabaceae	Water	AISI 304, Carbon steel and brass sheets	3.5% NaCl	20, 50	---	---	---	0.25	30–60–90 days
[90]	Glycine	Glycine	Dried	Fabaceae	Water	AISI 304, Carbon steel and brass sheets	3.5% NaCl	20, 50	---	---	---	0.25	30–60–90 days
[91]	*Lavandula angustifolia* L.	Lavanda	Oil	Lamiaceae	Ethanol	Al-Alloy 5754	3% NaCl	25, 40, 60	Langmuir	99	Cathodic	0.5	4 h
[92]	*Bagassa guianensis*	Amapa-rana	Dried	Moraceae	Ethanol	Zn	3% NaCl	25	Langmuir	97	Mixed	10	1 h
[93]	*Aerva lanata*	Mountain knotgrass	Petals	Amaranthaceae	Water	Al-Alloy AA 6061	3.5% NaCl	25	Langmuir	89	Mixed	---	1 h
[94]	*Artemisia annua* L.	Sweet wormwood	Stem	Asteraceae	Seawater	Al-Alloy 5083	Seawater	25	Freundlich	67	Anodic	0.5	1 h
[95]	*Carica pawpaw*	Pawpaw	Leaves	Caricaceae	Ethanol	Mild steel	3.5% NaCl	20	---	--	Mixed	---	21 days
[96]	*Nicotiana glauca*	Nicotiana	Dried leaves	Solanáceas	Water	Mild steel	0.1 and 1 M NaCl	30	---	86.5	Anodic	0.3	---
[97]	*Phoenix dactylifera* L.	Date Palm	Fruit juice	Arecaceae	Water	Al-Alloy 7075	3.5% NaCl	20	Temkin	63	Cathodic	1	---
[98]	*Camellia sinensis*	Tea Plant	Leaves	Theaceae	Water	Mild steel	Water and 35 ppm Al_2_(SO_4_)_3_ and 10 ppm Cl^−^	30, 40, 50	Langmuir	89.7	Mixed	1	21 h
[99]	*Lawsonia inermis*	Alheña	Commercial henna powder	Lythraceae	---	Al-Alloy 5083	Seawater	---	Langmuir	88	---	---	60 days
[100]	*Curcuma*	Curcum	Bark and rhizomes	Zingiberaceae	Methanol	Steel	3.5% NaCl + Absence and presence of Na_2_S 16 ppm	25	Temkin	77.4	Mixed	1	---
[101]	*Croton cajucara*	Sacaca	Powdered bark	Euphorbiaceae	Methanol	AISI 1020	0.5% NaCl	20	Langmuir and Frumkin	93.84 and 64.73	Mixed	0.8	---
[102]	*Myrmecodia pendans*	Myrmecodia	Plant material	Rubiaceae	Ethanol	Carbon steel API 5L Grade B	3.5% NaCl	25	---	84.82	Mixed	10	---
[103]	*Vernonia amigdalina*	Bitter leaf	Leaves	Asteraceae	Ethanol	Carbon steel	3.5 M NaCl	20	---	90.08	---	---	39 days
[104]	*Mansoa alliacea*	Garlic sacha	Leaves	Bignoniaceae	Ethanol	Zn	3% NaCl	25	Langmuir	90	Mixed	0.3	---
[105]	*Lawsonia inermis*	Henna tree	---	Lythraceae	Water	AISI 304L	3.5% NaCl	25	Langmuir	92.7	Cathodic	1	360 h
[105]	*Rosmarinus officinalis* L.	Romero/Rosemary	---	Lamiaceae	Water	AISI 304L	3.5% NaCl	25	Langmuir	92.7	Anodic	1	360 h
[106]	*Amaranthus cordatus*	Amaranto	Leaves	Amaranthaceae	Ethanol	Mild steel	0.5 M and 1 M H_2_SO_4_ and NaCl	20	---	99.51	---	---	30 days
[107]	*Clitoria ternatea*	Campanilla	Flowers	Fabaceae	Water, ethanol	Mild steel	3.5% NaCl	30	Langmuir	89.91	Mixed	0.	1 h
[108]	*Aloe vera*	Sábila	Leaves (Gel)	Xanthorrhoeaceae	---	Mild steel	1% wt NaCl	25		81.81	Mixed	10	2 h
[109]	*Pelargonium*	Pelargonios	Aerial parts	Geraniaceae	Water	Mild steel	1 M HCl	25	Langmuir	90.61	Mixed	0.5	6 h
[110]	*Azadirachta indica*	Neem	Leave	Meliaceae	Water	Mild steel	Salt water	---	---	~74	---	---	1500 h
[111]	*Cinnamomum zeylanicum*/*Cinnamomum verum*	Cinnamon tree	Inner bark	Lauraceae	Water	Al-Alloy	1% NaCl	60, 90	Langmuir	61.1	Mixed	1	1 h
[112]	*Myrmecodia pendans*	Myrmecodia	--	Rubiaceae	Ethanol	Carbon steel API 5L Grade B	3.5% NaCl	20	Langmuir	79.7	Mixed	100	---
[113]	*Olea europaea*	Olive	Leaves	Oleaceae	Water	Cu	0.5 M NaCl	25, 35, 45, 55	---	90	Cathodic	0.5	24 h
[114]	*Xanthosoma* spp.	Mafata/Otoe	Leaves	Araceae	Water	Cu	Seawater	30, 40, 50, 60	Langmuir	85	---	---	6 and 24 h
[115]	*Origanum majorana*	Mejorana/Mayorana	Leaves	Lamiaceae	Solvent	Mild steel	0.5 M m NaCl	30, 35, 40, 45	---	90	Mixed	10	2 h
[116]	*Nicotina*	Tabacco	--	Solanaceae	Water	Carbon steel Q235	Seawater	25, 45, 60	--	83.90	Mixed	0.57	--
[117]	*Phyllanthus muellerianus*	Phyllanthus	Leaves	Euphorbiaceae	Methanol	Carbon steel	3.5% NaCl	26	Langmuir	97.58	---	---	40 and 89 days
[118]	*Seaweed*	Seaweed	Commercial extract	Macroalgae	0.5 M NaCl	Carbon steel	Saline formation water	25, 35, 45, 55	Temkin	93	Anodic	0.5	24 h
[119]	*Eucalyptus globulus*	White eucalyptus	Leaves	Mirtáceas	Water	Steel	0.1 mol/L NaOH + 0.5 m/L NaCl	20	---	87.65	Mixed	1	---
[119]	*Punica granatum*	Pomegranate	Trunk	Lythraceae	Water	Steel	0.1 mol/L NaOH + 0.5 m/L NaCl	20	---	92.61	Mixed	1	---
[119]	*Olea europaea*	Olive	--	Oleaceae	Water	Steel	0.1 mol/L NaOH + 0.5 m/L NaCl	20	---	92.05	Mixed	1	---
[120]	*Capsicum* from chile	Chili peppers	---	Solanáceas	--	Carbon steel	3.5% NaCl	25	--	92	Mixed	1	240 h
[121]	*Vernovia amygdalina*	Bitter leaf	Leaves	Asteraceae	--	Al-Alloy	4 M NaCl	20	--	--	--	--	240 h
[122]	*Slanum nigrum*	Blackberry grass	Leaves and grains	Solanáceas	Methanol	Zn	3.5% NaCl and presence 16 ppm of Na_2_S	30, 35, 40, 45, 50	Langmuir and Freundlich	81.50	Mixed	1	180 min
[123]	*Commiphora myrrha*	Mirra	Sap	Burseraceae	Methanol	Brass	3.5% NaCl and presence Na_2_S 16 ppm	25, 35, 45, 55	Langmuir	67	Mixed	1	4 h
[124]	*Cascabela thevetia*	Yellow oleander	Leaves	Apocynaceae	Water	Carbon steel	3.5% NaCl and presence Na_2_S 16 ppm	25	Freundlich	95	Mixed	1	30, 60, 90, 120, 150 and 180 min
[125]	*Nigella sativa*	Absinthe	Seeds	Ranunculaceae	0.5 M NaCl	Mild steel	NaCl 0.5 M	20	Langmuir	91	Mixed	1	--
[126]	*Ilex kudingcha*	Kudingcha	Leaves	Aquifoliaceae	Ethanol	Carbon steel J55	3.5% NaCl with CO_2_	20	Langmuir	96.53	Mixed	1	--
[127]	*Centaurea cyanus*	Cornflower	Commercial extract	Asteraceae	--	Carbon steel	Saline formation water	10, 20, 30, 40	Langmuir	69.2	Mixed	2	2 weeks
[128]	*Pithecollobium dulce*	Guamúchil	Leaves	Leguminosas	Ethanol	Mild steel	1 M HCl and 3.5% NaCl	20	-- -	77	Mixed	---	7, 14, 21 and 28 days
[129]	*Matricaria recutita chamomile*	Castilla chamomile	Dried	Asteráceas	Water	Carbon steel S235JR	0.5 M NaCl	25, 35, 40, 45	Langmuir	98.90	Mixed	1	---
[130]	*Brassica campestris*	Yuyo	Aerial parts	Brassicaceae	Ethanol	Steel	3% NaCl	20	---	84	Anodic	1	---
[131]	*Green nettle*	Nettle	Leaves	Urticáceas	Water	Mild steel St-12	3.5% NaCl	---	---	95	Mixed	1	---
[132]	*Musa paradisiacal*	Banana	Peel	Musaceae	Methanol	Fe	3% NaCl	---	---	67.90	---	--	12 days
[133]	*Aerva lanata*	Bloodthirsty of Cuba plant	Leaves	Amaranthaceae	Water	Carbon steel	200, 250, 300 and 350 ppm NaCl	30, 40, 50, 60	---	95	---	---	7, 30 and 60 days
[134]	*Glycyrrhiza glabra*	Persian Licorice	Leaves	Fabaceae	---	Mild steel	3.5% NaCl	25	--	98.80	Mixed	0.5	72 h
[135]	*Gingko biloba*	Gingko	Fruits	Ginkgoaceae	Ethanol	Carbon steel J55	3.5% NaCl with CO_2_	20	Langmuir	95	Mixed	1	---
[136]	*Oryza sativa*	Rice	Straw	Poaceae	Ethanol	Carbon steel SAE1045	3.5% NaCl	25	---	92	---	---	42 days
[137]	*Pistacia terebinthus*	Pistacia	Leaves	Anacardiaceae	Methanol	Fe	3% NaCl	--	--	96.96	--	1	24 h
[138]	*Cocos nucifera*	Coconut	Coconut Shell powder	Arecaceae	---	AISI 904L	3.5% NaCl	20	Langmuir	99.99	Mixed	2	168 h
[139]	*Kalanchoe blossfeldina*	Kalanchoe	Ground fine powder of plant	Crassulaceae	Water	Carbon steel	Polluted NaCl (3.5% NaCl + 16 ppm Na_2_S)	29.85	Temkin	84	Mixed	1	180 min
[140]	*Cellulose niacin* nano-composite	--	Ethyl Cellulose niacin composite	--	Methanol	Cu	3.5% NaCl	25	--	94.7	Mixed	1	1 h
[141]	*Averrhoa bilimbí linn*	Wuluh Starfruit	Leaves extract	Oxalidaceae	Ethanol	Reinforced steel	3% NaCl	25	-	99.7	-	-	14 days
[142]	*Olea europaea*	Olive	Olive leaf	Oleaceae	Water	Cu	0.5 M NaCl	25	--	95		-	24 h
[143]	*Aminotriazolethinol*-functionalized chitosan	Chitosan	--	--	Water	Stainless steel	3.5% NaCl	25	Langmuir	97.8	mixed	1, 10, 50 and 100	-
[144]	*Ammi visnaga* (L.) Lam	Khella	Essential oil	Apiaceae	---	Brass	3% NaCl	25	---	95.65	---	---	---
[145]	*Jatropha curcas*	Jatrofa	Extracts powder	Euphorbiaceae	Wáter	Mild steel	3.5% NaCl	25	---	90.26	---	0.3	8 weeks
[146]	*Areca catechu*	Betel tree	---	Arecaceae	Methanol	Mild steel	0.5 M NaCl	28	Langmuir and Temkin	50	Anodic	---	240 h
[146]	*Laurus nobilis*	Bay tree	---	Lauraceae	Methanol	Mild steel	0.5 M NaCl	28	Langmuir and Temkin	65	Mixed	---	240 h
[146]	*Catharanthus roseus*	Bright eyes	---	Apocynaceae	Methanol	Mild steel	0.5 M NaCl	28	Langmuir and Temkin	82	Anodic	---	240 h
[147]	*Natural polymers*	---	---.	---	Water	Mg-Alloy	3.5% NaCl	25	---	80.56	---	0.2	24 h
[148]	*Piper nigrum*	Piper	Fruit powder	Piperaceae	Ethyl alcohol	Carbon steel	CO_2_–saturated 3.5%NaCl	25	Langmuir	98.9	Mixed	1	---
[149]	*Cyamopsis tetragonoloba*	Guaran	Guar gum–grafted methyl methacrylate (GG–MMA)	Fabáceas	Water, ascorbic acid, methyl methacrylate and potassium persulfate	Carbon steel P110	CO_2_–saturated 3.5%NaCl	50	Langmuir	96.8	Cathodic	0.1	6 h
[150]	*Cucumeropsis mannii*	Egusi	Shells	Cucurbitaceae	Ethanol	Carbon steel A515 Grade 70	1 M NaCl	30	Langmuir	91.2	Mixed	0.3	1, 3, 5, 7 days
[151]	*Olea europaea*	Olive	Leaves extract	Oleaceae	Methanol, Ethyl acetate, dichloromethane and Hexane	Carbon steel	NaOH (0.1 M) + NaCl (0.5 M)	25	---	91.9	Mixed	1	---
[152]	*Malus domestica*	Apple tree	Apple pomace-derived	Rosaceae	Ethanol	Carbon steel SAE 1010	3.5% NaCl	---	Langmuir	98.8	---	---	7 days
[153]	*Lawsonia inermis* extract	Henna	Dried Powder	Lytharaceae	Water	AISI 4130	3.5% NaCl	50	---	90.14	---	---	---
[154]	Cinnamomum	Cinnamon	Cinnamaldehyde	Lauraceae	Methanol and Water	Mild steel	3% NaCl	25	Langmuir	70	Mixed	---	1 week
[155]	*Citrus maxima*	Grapefruit	Peel	Rutaceae	Ethanol	Carbon steel	3.5% NaCl	30	Langmuir	71.15	Mixed	1	6 week
[156]	*Citrus* × *sinensis*	Orange	Orange peel	Rutaceae	Ascorbic acid, naringin, and neohesperidin	Mg-Alloy AZ91D	0.05 wt% NaCl	---	Langmuir	85.7	Mixed	10	36 h
[157]	Ethanolic extract propolis (EEP)	Propolis	---	--	Ethanol	Carbon steel SAE 1010	3.5% NaCl	---	Langmuir	79	Mixed	1	6 h
[158]	*Ipomoea batatas* L. and curcuma longa	Purple sweet potato and turmeric extract	---	Convolvulaceae and Zingiberaceae	---	Carbon steel API-5L	3.5% NaCl	---	---	82.54	Mixed	---	12 days
[159]	*Davidian involucrata*	Davidia	Leaves	Cornaceae	Water	Carbon steel Q235	0.2 M NaOH, 0.5 M KOH, 0.3 M Ca(OH)_2_ and 3.5 wt%CaCl_2_	35	---	96	Mixed	1	18 h
[160]	*Salvia officinalis* L.	Dalmatian sage	---	Lamiaceae	Petroleum ether	Sn	3% NaCl	25	---	94.1	Mixed	1	2.5 h
[161]	*Solanum melongena*	berenjena	Stem (brinjal cap)	Solanáceas	Water	Mild steel	1 M NaCl	---	---	74	Mixed	5	24 h
[162]	*Prunus persica*	Peach pomace	Peach pomace	Rosaceae	---	Mild steel	0.5 M NaCl	---	---	88	---	---	48 h
[163]	*Azadirachta indica*	neem	Leaf	Meliaceae	Water	Carbon steel	3.5% NaCl	0, 50	---	95	---	---	182 days
[164]	*Pinus resinosa*	pino rojo americano	Conifer cone	Pinaceae	Water	Steel	(NaOH), (KOH), (Ca(OH)_2_ and (NaCl)	25	Langmuir, Freundlich and Temkin	81.2	---	0.1	720 h
[165]	*Cinnamomum verum*	Cinnamomun trees	Extracted Cinnamon essential oil	Lauraceae	Water	Cu	3 wt% NaCl	25	---	89	Cathodic	0.2	16 h
[166]	*Skytanthus acutus* meyen	Cacho de cabra	Leaf	Apocynaceae	Water	Carbon steel AISI1020	0.5 M NaCl	---	---	90	Mixed	2	48 h
[167]	*Treculia africana*	African breadfruit	Leaves	Moraceae	Etanol/water	Al-Alloy AA7075-T7351	2.86% NaCl	25	Langmuir, Freundlich and Temkin	91	Anodic	1	7 h
[168]	*Ocimum basilicium*	Albahaca	Seeds	Lamiáceas	Water	Al-Alloy 2024-T3	3 wt% NaCl	25	Langmuir	95.5	Mixed	1	6 h
[169]	Chitosan derivative with titanium (CPT)	Quitosano	---	--	4-PA and CS	Al-Alloy C3003	3.5 wt%	25	Langmuir	94.5	Mixed	1	72 h
[170]	*Pistacia lentiscus*	Lentisco	Leaves	Anacardiaceae	Water	Ti-Alloy	3 wt% NaCl	25	Langmuir	84.58	---	---	24 h
[171]	*Lawsonia inermis* L.	Henna	Leaves	Lythraceae	Ethanol	Carbon steel	0.5 M NaCl	25	Langmuir	93	Mixed	0.5	14 days
[172]	*Syn. Aloe maculata*	Aloe saponaria tannin	---	Asphodelaceae	Acetone/water	Bronze B66	3% NaCl	---	---	90	---	1	24 h
[173]	Ce-citrate	Ce-citrate	---	---	---	AISI 4130	0.05 M NaCl	25	---	94	---	1	96 h
[174]	*Vicia faba*	Haba	Peel	Fabaceae	Hexane	Mild steel	3.5% NaCl	25	Langmuir, Freundlich and Temkin	97.84	Mixed	1	24 h
[175]	*Ficus pumila* Linn	Creeping fig	Leaves	Moraceae	Methanol/Water (70:30)	XC38 steel	0.1 M NaCl	20	---	42.4	Mixed	0.5	2 h
[176]	*Fucus vesiculosus*	Sargazo vejigoso	Soft section	Fucaceae	Ethanol/Water (80:20)	AISI 304	3.5% NaCl	25	Langmuir	81.7	Mixed	---	4 days
[177]	*Pistacia lentiscus* L.	Lentisk	Leaves	Anacardiaceae	Ethanol	Fe	3% NaCl	25	---	82	Cathodic	1	30 min

Although the mechanisms are not entirely clear, owing to the wide variety of organic groups that can promote corrosion inhibition, researchers have focused on studying the potential mechanisms in detail. Lai et al. [178] have studied the influence of methionine on the corrosion behavior of twinning-induced plasticity steel in 3.5% NaCl. Using Raman spectroscopy, the researchers concluded that the corrosion peaks decreased with increasing methionine concentration because of its adsorption on the surface, along with a mixed inhibition mechanism. Gadow et al. [123] have studied the inhibitory effects of myrrh extract on the corrosion of brass in a 3.5% NaCl solution contaminated with 16 ppm sulfide. It is well known that the sulfide present in the marine environment promotes the corrosion of copper by forming CuS. The researchers found that the corrosion rate decreased with increasing concentrations of the myrrh extract. In addition, using the Langmuir method and adsorption isotherms, the authors described the adsorption mechanism as physisorption. To search for green alternatives to inhibit corrosion, Othman et al. [136] have studied the inhibition of steel corrosion in 3.5% NaCl using rice straw extract, which was exposed to immersion for 42 days in a NaCl solution in the presence of the inhibitor. Rice straw extract is mainly composed of low-cost industrial polymers and is rich in lignocellulose and lignocarbohydrate complexes. Owing to the characteristics of these polymers, researchers indicated that they were able to inhibit corrosion for 42 days, achieving 92% efficiency compared to commercial lignin. Furthermore, the formation of complex organometallic compounds with polymers was demonstrated, which contributed to the inhibition of corrosion. Othman et al. proposed that the inhibitory mechanism involves interphase inhibition, which involves the formation and stabilization of oxide–hydroxide layers on carbon steel surfaces. This combined reaction, involving steel oxidation, organometallic complex formation, and adsorption processes, significantly contributed to the effective corrosion inhibition of low-carbon steel.

In this line, there are extensive studies on plant-based green corrosion inhibitors. Table 1 shows the wide range of plant species that have been used in laboratory tests. *Rosmarinus officinalis* [66,105], commonly known as rosemary, shows a particular adhesion to the surface of the Al-Mg alloy and AISI 304 L, generating cathodic inhibition efficiency (IE) of 93.8% and 92.7% in contact with a 3% to 3.5% NaCl saline solution at 25 °C, 30 min and 360 h, respectively. The adsorption behavior on the surface of the Al-Mg alloy follows the Freundlich isotherm over a 30 min exposure period [66]. On the other hand, the plant species *Ambrosia maritima*, commonly known as damsissa [65,73,89], exhibits an anodic IE of 65% on common steel in contact with a 0.5 M Na_2_SO_4_ solution with different concentrations of NaCl, as determined from EIS measurements within a frequency range of 10 kHz to 100 mHz.

Furthermore, the same plant species showed similar behavior when in contact with metals such as Zn [73,92,104,122] and Al [167,168,169] in 0.5 M NaCl, achieving a mixed IE of 90.7% and 88.3%, respectively. The species *Lupinus* [65,73] was analyzed using both (i) the dry plant and (ii) seeds in contact with Zn and common steel in 0.5 M Na_2_SO_4_ with 0.5 M NaCl. The results of these studies show promising corrosion inhibition properties. The dry plant material showed an IE of approximately 68% for common steel and 89.1% for the Zn surfaces using seeds. Both natural products were analyzed according to the Langmuir adsorption isotherm and followed anodic and mixed inhibition at a scan rate of 0.35 mV/s. Similarly, the seeds of *Cymbopogon proximus*, commonly known as Halfabar [73], also show an excellent mixed inhibition efficiency of 94.7% for Zn in contact with a 0.5 M NaCl solution, following the Langmuir isotherms for 1 day, respectively. A plant species named *Ammi Visnaga* revealed a high corrosion inhibition of approximately 93.04% for the Cu-Zn alloy in contact with a 3% NaCl solution at 25 °C, which was evident even after interacting for 60 min with the solution [75]. Electrochemical studies were conducted under conditions where the scan rate was increased to 0.5 mV/s within a frequency range of 10 kHz to 10 mHz for 60 min, compared to other experiments.

The efficacy of *Catharanthus roseus*, or *Vinca rosea*, shows a mixed inhibition of 70% in protecting mild steel in contact with a 3.5% NaCl solution at 35 °C [79,146]. Additionally, carbon steel A106 was studied in contact with honey, obtaining an anodic inhibition of approximately 91.23% in saline water [82]. In both cases, the study lasted for 5 days. Furthermore, *Ricinus communis*, or castor, showed notable inhibition efficiency using extracts from both leaves in ethanol and fruits in methanol [86] after 120 days of operation. The leaves exhibited a mixed inhibition with an IE of 84% on mild steel in contact with 100 ppm NaCl at 20 °C, whereas the fruits showed a mixed corrosion inhibition IE of 79% on common steel in 3.5% NaCl. Similarly, *Hibiscus rosa-sinensis Linn*, or China rose, showed an extraordinary inhibition efficiency of 98% on carbon steel in contact with 60 ppm Cl^−^ ion solutions in the presence of Zn^2+^ ions, using water as the solvent [88] in a shorter contact time of approximately 1 day.

Other plant extracts, such as *Camellia sinensis* [90,98] have been applied to mild steel, carbon steel, AISI 304, and brass, giving EI values of approximately 89.9% after an exposure time of 90 days in 3.5% NaCl. Furthermore, *Lavandula angustifolia*, known worldwide as lavender, which was used as a corrosion inhibitor for the Al-3 Mg alloy in 3% NaCl, showed inhibition efficiency (IE) values of almost 99% at temperatures between 25 and 60 °C, and the adsorption behavior could be described by the Langmuir adsorption isotherm [91]. Zn protection was tested on dried *Bagassa guianensis*, demonstrating a cathodic EI of approximately 97% after 1 h of contact [92].

Other plant-derived species, such as *Artemisia annua* L., are used to protect metal surfaces in saline aqueous environments. It was evaluated as an inhibitor of the aluminum alloy Al 5083 in natural seawater, obtaining an anodic EI of 67% after an exposure time of 1 h. The adsorption mechanism on the surface of Al-5083 follows the Freundlich isotherm [94]. On the other hand, *Phoenix dactylifera* L., as a corrosion inhibitor of the Al-7075 alloy, gave a cathodic IE value of approximately 63%, following a Temkim adsorption isotherm [97]. *Amaranthus cordatus* generated an impressive EI of approximately 99.51% after 30 days of contact with the NaCl solution in the presence of the inhibitor [106]. Other green corrosion inhibitors such as *Vernonia amygdalina* generate 90.09% corrosion inhibition in carbon steel after 39 days of exposure [103]; *Commiphora myrrha* shows a corrosion inhibition efficiency of 67% for brass after 4 h of contact [123]. *Cascabela thevetia* [124] and *Nigella sativa* [125] show corrosion inhibition efficiencies of 67 and 95%, respectively. These inhibitors generally exhibit varied inhibition efficiencies and adsorption behaviors for different steels. They are adsorbed on metal surfaces following the Langmuir and Freundlich adsorption isotherms for the inhibition of mixed or anodic corrosion.

The mechanisms by which different plant species inhibit corrosion are complex. According to studies on the inhibitory process, inhibition actions are multifunctional (cathodic, anodic, or mixed). Additionally, inhibition efficiency changes significantly depending on the origin of the plant material and the type of metal to be protected. Other factors that also influence inhibition efficiency are the method of extracting the inhibitory species from the plant, the exposure time to the solution containing the inhibitor, the temperature, and the experimental conditions used during the measurement of the inhibitory action. Many methodologies presented in the literature vary substantially. However, these findings highlight the importance of investigating green inhibitors as an alternative to conventional ones because they offer a sustainable approach to preserve the mechanical integrity of the metal and increase the useful life of industrial facilities.

## 5. Typical Electrochemical and Surface Characterization Techniques

Electrochemical and surface characterization techniques play crucial roles in evaluating the performance and effectiveness of environmentally friendly corrosion inhibitors. These methods offer valuable information about the inhibitory properties of organic compounds extracted from plant materials and help understand their interactions with metal surfaces when they are in contact with corrosive environments. Below is a list of electrochemical and surface characterization techniques commonly employed in the evaluation of green corrosion inhibitors.

### 5.1. Potentiodynamic Polarization (Tafel Analysis)

**Figure 7:**
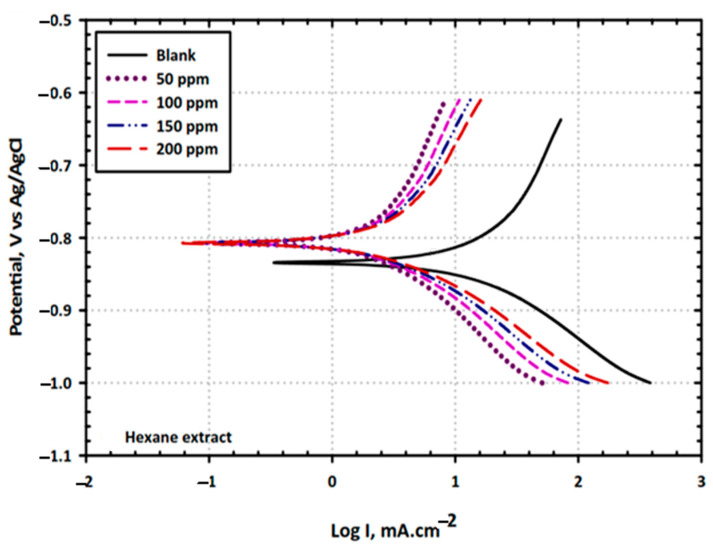
Potentiodynamic polarizations for mild steel in 3.5% NaCl solution in the absence and presence of various concentrations of hexane extract of *Vicia faba* at 298 K [174].

This electrochemical technique is widely used to measure the corrosion rate of metals in the absence and presence of inhibitors. This technique allows the determination of the kinetic parameters associated with the corrosion process, such as (i) the anodic and cathodic exchange current densities (i_0_), (ii) anodic and cathodic Tafel slopes (β), (iii) corrosion potential (E_corr_), and (iv) corrosion current density (i_corr_) (see Figure 7). Tafel analysis, derived from the simplification of the Butler–Volmer equation, is a widely applied technique for determining the corrosion current and potential. However, this technique is limited to small overpotentials (>±118 mV), where a pure charge transfer control will dominate the electrode process, and the contribution of the backward reaction in a redox reaction can be negligible (~1%) to the overall current [179,180]. The results obtained with this technique help to understand the efficiency of green corrosion inhibitors, specifically when the metal is in contact with a saline electrolyte and the anodic and cathodic reactions begin to proceed. Finally, it is important to determine the extent to which the useful life of a metallic material can be prolonged using corrosion inhibitors. Figure 7 shows potentiodynamic polarization curves for mild steel in 3.5% NaCl solution in the presence and absence of various concentrations of hexane extract of *Vicia faba*. The results indicate that the inhibitor effectively decreases the corrosion rate, acting as a mixed-type inhibitor with predominant control of the anodic reaction [174].

To understand the corrosion mechanism, Alibakhshi et al. [134] have recorded polarization curves for mild steel samples after an immersion time of 24 h with and without various concentrations of Persian licorice (Table 2). The corrosion current density (i_corr_), anodic Tafel slope (βa), cathodic Tafel slope (−βc), and corrosion potential (E_corr_) were obtained using the Tafel extrapolation method. Their results confirmed a mixed-type inhibitor in NaCl solution, where the shifting of E_corr_ suggests a high inhibitor role on the anodic dissolution reaction mechanism of the steel. Consistent with the other electrochemical measurements, the polarization data showed that 600 ppm of Persian licorice provided the best corrosion inhibition performance, highlighting its potential to reduce the corrosion rate of mild steel through adsorption and/or film formation on the active sites.

### 5.2. Electrochemical Impedance Spectroscopy (EIS)

**Figure 8:**
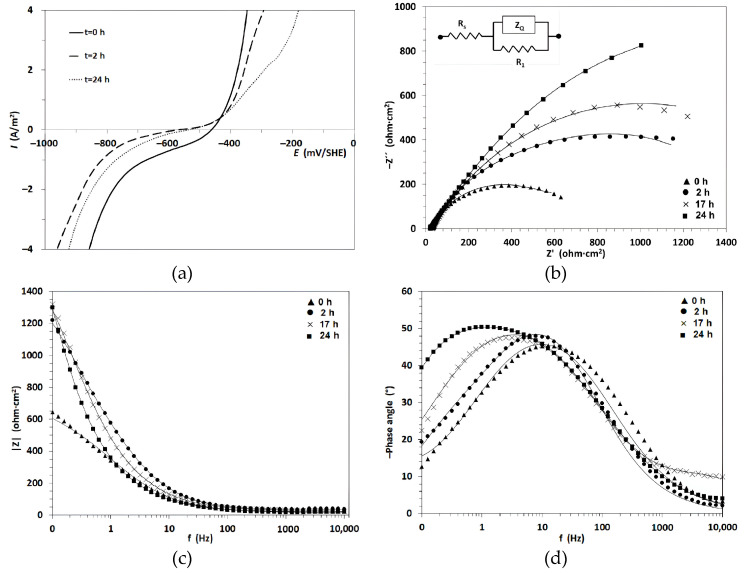
Electrochemical results for carbon steel in 0.5 M NaCl + 600 ppm DR (N_2_) at different immersion times: (**a**) polarization curves; (**b**) Nyquist diagram; (**c**) Bode magnitude diagram; and (**d**) Bode phase angle diagram. Marks and continuous lines in EIS diagrams indicate the experimental and fitted data, respectively [166].

EIS is a non-destructive electrochemical technique that allows characterization of the response of a metal–electrolyte interface during the application of alternating current (AC), and consequently, how this response varies in the presence of an inhibitor. It provides information on (i) charge transfer resistance, (ii) double-layer capacitance, and (iii) diffusion processes on the metal surface. Furthermore, by adjusting the impedance results reflected in the Nyquist and Bode diagrams, it is possible to emulate the behavior of the interface with that of an electrical equivalent circuit (see Figure 8). EIS is a valuable technique for studying the inhibition mechanism and film formation behavior of green corrosion inhibitors under steady-state conditions performed over a wide range of frequencies. Figure 8 shows polarization and EIS measurements for carbon steel in 0.5 M NaCl in the presence of 600 ppm of Skytanthus dry extract under different immersion times. According to the Nyquist diagram, the spectra indicate a single semicircle that increases with the contact time of the sample in the test solution, suggesting the formation of a protective layer on the steel [166].

### 5.3. Scanning Electron Microscopy (SEM), Energy-Dispersive Spectroscopy (EDS)

**Figure 9:**
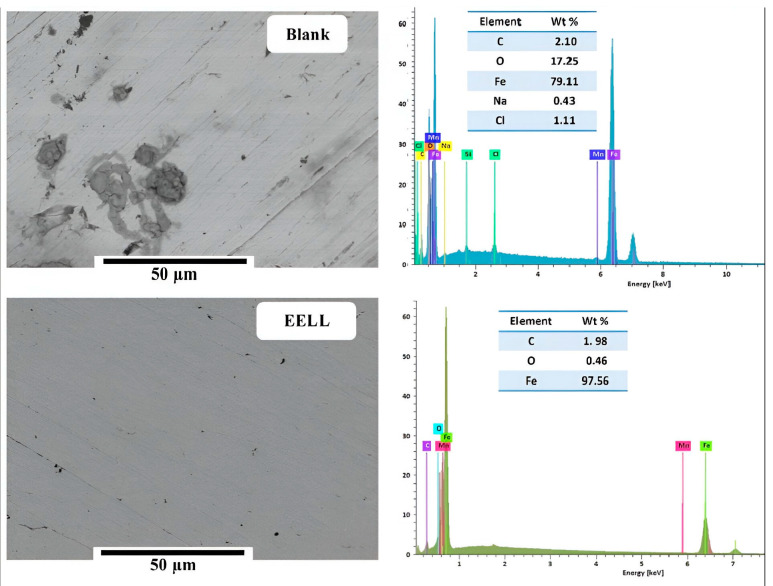
SEM/EDS micrographs of iron after immersion for 24 h in 3% NaCl solution without (Blank) and with 100 ppm ethanol extract of *Lentisk leaf* (EELL) [177].

SEM-EDS equipment represents a pinnacle of technological expertise, offering unparalleled specificity and power in material surface characterization. It is a basic tool in the research of various materials, particularly special metals that undergo corrosion. These images, captured at an extraordinarily high resolution, serve as invaluable windows in the intricate world of corrosion processes. They illuminate the spectrum of corrosion products formed and reveal the intricate dance of inhibitory films that coat the metal surfaces. Beyond mere observation, SEM-EDS help decipher the surface morphology and elemental distribution in the layer of the formed corrosion products (see Figure 9). This unravels the delicate interplay of molecular adsorption, revealing that the formation and adhesion of thin organic films are crucial to inhibiting corrosion. By examining the depths of these images, it is possible to gain insights into the mechanisms that govern the degradation or protection of metal surfaces. In doing so, SEM-EDS become not just a tool, but a gateway to understanding and shaping the fate of materials in their battle against corrosion. At the same time, energy dispersive analysis combined approach allows us to extract a richer trove of information, shedding light on the intricate chemical composition of these crucial barriers that protect against corrosion. Figure 9 shows SEM and EDS analysis for iron after immersion for 24 h in 3% NaCl with and without ethanol extract of *Lentisk leaf* (EELL). According to the Figure 9, in the black solution, the SEM/EDS analysis shows higher amounts of C, O, Fe, Na, and Cl, suggesting the formation of metal oxides/hydroxydes. However, in the presence of 100 ppm EELL, the SEM/EDS results show a remarkable reduction in the O amounts, indicating the adsorption of EELL on the metal surface, which prevents the formation of oxides/hydroxides [177].

### 5.4. Fourier Transform Infrared Spectroscopy (FTIR).

**Figure 10:**
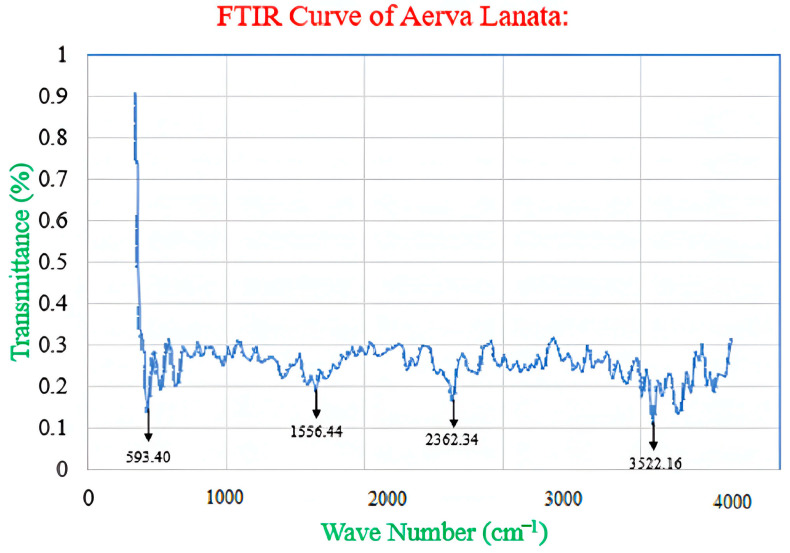
FTIR results of *Aerva lanata* [93].

Fourier Transform Infrared Spectroscopy (FTIR) plays a pivotal role in delineating the functional groups and chemical bonds initiated by green corrosion inhibitors during the adhesion process on metal surfaces. Additionally, FTIR facilitates a comprehensive understanding of various aspects: (i) elucidating the nature of the inhibitor, (ii) discerning its interactions with the metal surface, and (iii) precisely identifying the structural nuances and chemical bonds inherent to green corrosion inhibitors. Figure 10 reports the FTIR spectra for *Aerva lanata* flower extract used as corrosion inhibitor for carbon fiber-reinforced aluminum laminate in 3.5% NaCl. The spectra show bonds O–H, C–Cl, C–O and C–H, causing peak centers at 3522.16, 2362.34, 1556.44, and 593.40 cm^−1^ belonging to the amine, carbolic–acid, alkene, and aromatic functional groups, respectively [93].

Various techniques have been employed to explore the anticorrosive properties of these inhibitors. Alibakhshi et al. [134] have investigated the use of Persian licorice to protect mild steel from corrosion in NaCl solutions. They characterized Persian licorice by identifying different functional groups using FTIR, suggesting that these groups might help to mitigate corrosion; a coordination of the Persian licorice to iron on the mild steel surface is proposed. To evaluate corrosion, the researchers studied the influence of the inhibitor in 3.5 wt% NaCl using EIS and polarization measurements. EIS results revealed that the sample exposed to the solution without the inhibitor exhibited a slight decrease in low-frequency impedance over time, along with a decrease in the high-frequency phase angle plateau. This behavior can be attributed to the onset of corrosion and the gradual formation of corrosion products on the surface, which provides some level of protection against the continued dissolution of mild steel. This effect increased with the quantity of inhibitor up to 600 ppm, demonstrating that Persian licorice is an excellent corrosion inhibitor.

### 5.5. X-ray Photoelectron Spectroscopy (XPS)

**Figure 11:**
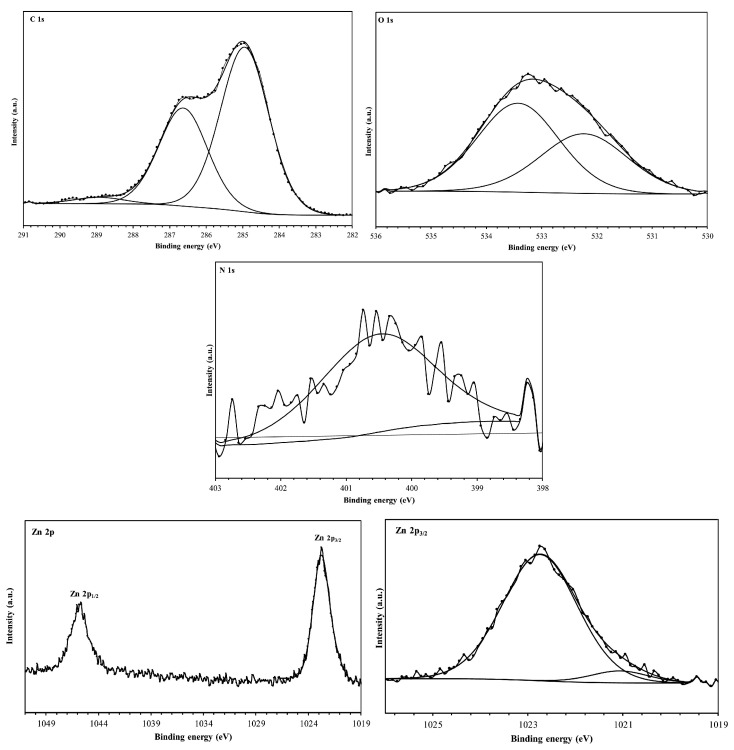
C 1s, O 1s, N 1s, and Zn 2p XPS deconvoluted profiles for zinc surface treated with *Bagassa guianensis* plant extract in 3% NaCl at 25 °C [92].

X-ray Photoelectron Spectroscopy (XPS) stands as a highly sophisticated and surface-sensitive technique that offers profound insights into the studied surfaces. By meticulously analyzing the elemental chemical composition and oxidation states of the surface-bound elements, XPS revealed the elemental composition of the metal surface (see Figure 11). Its utility extends notably to probing the intricate interactions between corrosion inhibitors and metal atoms, providing invaluable data that are crucial for understanding corrosion inhibition mechanisms.

According to Wang et al. [116], who characterized tobacco rob extract (TRE) using XPS analysis for corrosion studies of Q235 steel, a single peak was observed for both the O1s and N1s spectrums, which were attributes to the C–O bond from carbohydrate compounds and the C–N=C bond in pyridine rings originated from nicotine, respectively. In C1s spectrum, three main peaks were observed: the first was attributed to C–H, C–C and C=O bonds from TRE molecules; the second was ascribed to C–O bond (alcoholic hydroxyl and ether) in carbohydrate compounds; and the third was ascribed to the carbon atoms bonded to nitrogen C=N in the pyridine ring. The corrosion studies demonstrated inhibition efficiencies up to 83.9% in artificial seawater, which were attributed to the nicotine in TRE.

### 5.6. Atomic Force Microscopy (AFM)

**Figure 12:**
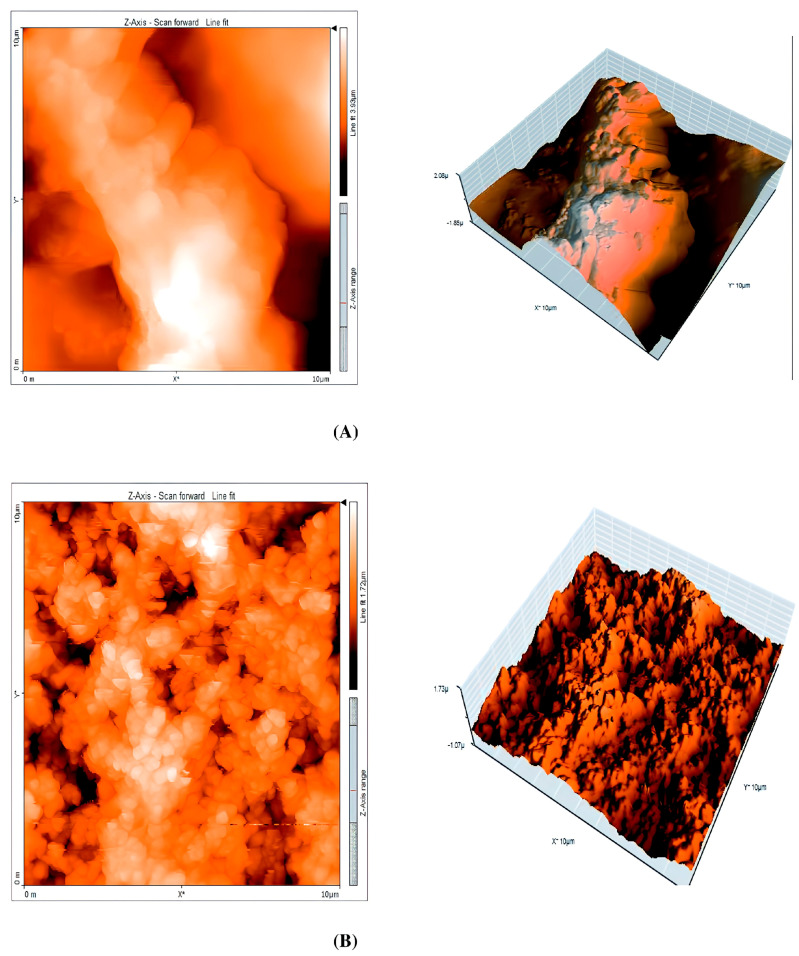
AFM 2D and 3D micrographs for α-brass electrode after 24 h immersion in 3.5% NaCl polluted by 16 ppm of sulfur ions (**A**) without and (**B**) with 300 ppm myrrh extract [123].

Atomic Force Microscopy (AFM) is a powerful technique that enables the acquisition of high-resolution images and provides invaluable insights into the topography and surface roughness of materials (see Figure 12). Its application extends notably to the examination of metal-inhibitor interfaces and is a valuable tool for investigating the adhesion dynamics and mechanical characteristics of inhibitor films on metal surfaces.

**Figure 13:**
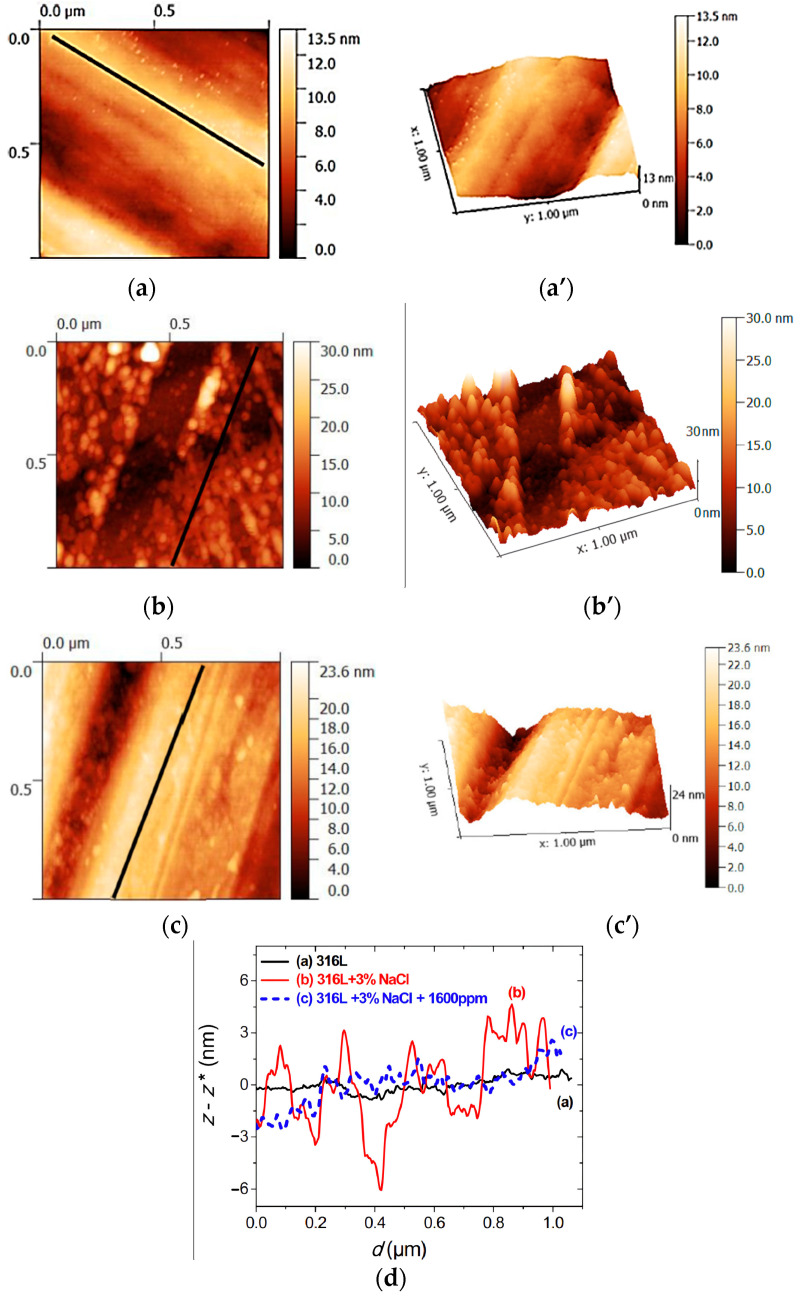
Two-dimensional and three-dimensional atomic force micrographs of 316L stainless steel: (**a**,**a′**) before immersion (polished), (**b**,**b′**) after immersion in 3% NaCl, and (**c**,**c′**) after immersion in 3% NaCl + 1600 ppm *Thymus satureoides* during 24 h, (**d**) calculated roughness (z-z*) factor for 316L stainless steel: (**a**) before immersion (polished), (**b**) after immersion in 3% NaCl, and (**c**) after immersion in 3% NaCl + 1600 ppm *Thymus satureoides* inhibitor during 24 h [181].

Most studies have focused on characterizing the surface before and after corrosion using the techniques. Simescu-Lazar [181] has studied the corrosion protection of 316 L stainless steel in 3% NaCl using the essential oil of *Thymus satureoides*. AFM and SEM were used to observe the surface of the steel specimens after immersion for 24 h in 3% NaCl, both with and without the inhibitor. Figure 13 shows the 2D and 3D images of the surface topography for 316L stainless steel after 24 h in 3.5% NaCl with and without thymus saturoides oil as a corrosion inhibitor. These AFM images show the rough surface of 316 L stainless steel immersed in 3% NaCl and a reduction in the surface roughness in the presence of the inhibitor. Figure 13 also displays the calculated difference between the roughness (z) and mean roughness factor (z*), denoted as (z-z*), corresponding to a profile from the respective 2D images. It was observed that the roughness profile of the steel surface immersed in 3% NaCl solution increase.

**Figure 14:**
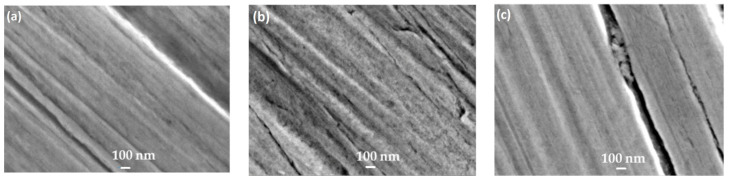
SEM image of 316L stainless steel: (**a**) before immersion (polished), (**b**) after immersion in 3% NaCl, and (**c**) after immersion in 3% NaCl + 1600 ppm inhibitor during 24 h [181].

Additionally, the samples were observed using SEM after immersion for 24 h. The surface of the polished stainless-steel sample was very smooth and showed no corrosion, whereas the stainless steel dipped in 3% NaCl without the inhibitor was very rough (Figure 14). However, the presence of the 1600 ppm inhibitor significantly decreased the rate of corrosion and surface damage, suggesting the formation of a protective inhibitor film on the 316 L stainless steel surface (Figure 14).

## 6. Theoretical Simulations by DFT

The efficacy of a green inhibitor depends on the adsorption of its molecular constituents on the metal surface. Consequently, the effectiveness of plant-derived natural extracts correlates intricately with the electronic and spatial molecular structures of their elemental components. Given that these components are complex mixtures of diverse phytochemicals, identifying the primary contributor to corrosion inhibition poses a challenge. To address this issue, quantum chemical calculations have emerged as a compelling and invaluable theoretical tool. These calculations enabled the dissection of discrete contributions from each molecule within the natural extract [182].

Although commercial corrosion inhibitors have historically safeguarded metals against corrosion, concerns regarding their environmental impact are large within the scientific community. Quantum chemical studies focus on the fundamental tenets of quantum mechanics, a branch of physics that delineates the behavior and interactions of subatomic particles [183].

This theoretical framework facilitates a nuanced understanding of molecular dynamics and aids in the design of environmentally sustainable corrosion protection strategies. In the context of materials science and corrosion research, quantum analysis allows scientists to probe the electronic structures, energetics, and bonding characteristics of molecules and materials at the quantum level. Through computational simulations and modeling, quantum analysis provides a detailed understanding of the fundamental processes governing corrosion inhibition.

Density Functional Theory (DFT) has proven instrumental in analyzing the characteristics of inhibitor/surface interactions and elucidating the structural dependence of the inhibitor on the corrosion inhibition process. Furthermore, DFT allows the calculation of several molecular parameters that are directly associated with the extract inhibition efficiency. This enables researchers to calculate electronic properties, predict molecular structures, and evaluate the adsorption energies of green inhibitors on metal surfaces. DFT simulations offer valuable insights into the stability and reactivity of inhibitor–metal interfaces [79,148,183].

**Figure 15:**
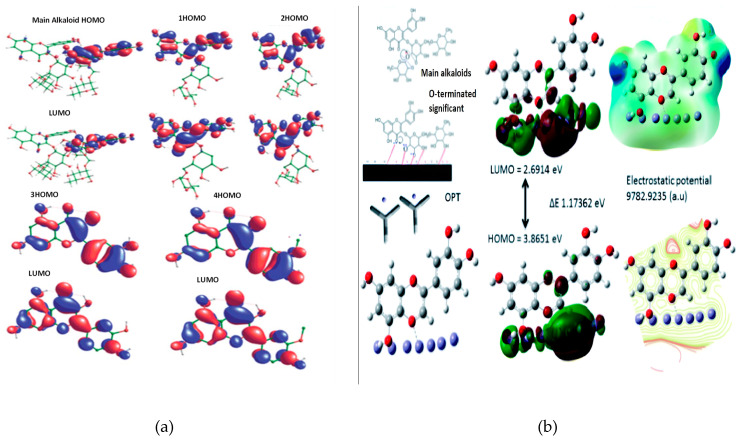
(**a**) HOMO and LUMO surfaces of neutral main alkaloids of *C. roseus* and (**b**) scheme of *C. roseus* extracts adsorbed on 111 mild steel surface [79].

The application of the frontier molecular orbital theory enables us to anticipate the interaction centers of the inhibitor molecules with the steel surface. Organic molecules can serve both as electron donors to vacant d-orbitals of iron and as electron acceptors. The energy of the highest occupied molecular orbital (EHOMO) is commonly linked to the electron-donating capacity of the molecule, whereas the energy of the lowest unoccupied molecular orbital (ELUMO) is associated with its propensity to accept electrons. Nars et al., [129] have founded that the highest occupied molecular orbital (HOMO) of the molecules presents in *Matricaria recutita* extract was mainly localized on the conjugated double bonds of the aromatic rings or those present in the other rings. The triple bonds of the carbon present in HDE and the single bond of carbon–azote in the molecule Pyrrollo [2,1-a] isoquinoline (PIS) are also involved in the HOMO. The lowest unoccupied molecular orbital (LUMO) was mainly occupied by carbon atoms engaged in the double bonds of the aromatic or other rings. In addition, electrophilic sites were identified on oxygen atoms. To evaluate the electronic interaction of the *Matricaria recutita* extract molecules with the metal surface, the authors have calculated quantum chemical parameters. The values of the estimated parameters for each extract constituent, such as global energy (E), dipole moment (µ), EHOMO, ELUMO, and the energy band gap (ΔE = ELUMO − EHOMO) were discussed [129]. Examples of the HOMO and LUMO for a molecule and its interaction with the metallic surface are shown in Figure 15.

Polarization studies have revealed that the inhibitor functions as a different type of inhibitor, leading to modifications in both the anodic and cathodic polarization curves. Specifically, cathodic inhibitors are effective in hindering cathodic reactions on metal surfaces, thereby inhibiting hydrogen evolution in acidic solutions and reducing oxygen in neutral or alkaline solutions. On the other hand, anodic inhibitors work by reducing anodic reactions and actively supporting the natural passivation of the metal through film formation, particularly in the pH range of 6–10. These anodic and cathodic inhibitors, often of organic origin, are adsorbed onto the metal surface, forming a protective barrier against dissolution at the anode and impeding hydrogen evolution or oxygen reduction at cathodic sites. Such inhibitors are known to be mixed-type inhibitors. The effectiveness of green inhibitors is intricately linked to their ability to adsorb and form films on the metal surfaces. This capability is influenced by several factors, including the molecular structure of the inhibitor, surface charge of the metal, and characteristics of the saline electrolyte. Green inhibitors that operate through multiple mechanisms offer metal protection through three primary avenues: physical adsorption, chemisorption, and the creation of an organic film on the metal surface. This protective coating acts as a barrier, shielding the metal from direct contact with the electrolyte and effectively thwarting corrosion, thereby ensuring long-term durability of the metal [81].

Such information will help in designing and tailoring green inhibitors for improved performance and effectiveness. Moreover, quantum analysis allows for the prediction of structure–activity relationships, providing guidance in selecting and optimizing green corrosion inhibitors. It also aids in predicting the efficiency of corrosion inhibitors under various environmental conditions, contributing to their practical application in real-world corrosion prevention scenarios.

## 7. Corrosion Testing Standards and General Guidelines for Corrosion Inhibitor Selection

For the reason of quality assurance, the industry has always tended to standardize material and their applications. Accordingly, in the case of metal corrosion, there are many standard tests that apply to specific metallic materials under different industrial situations [184]. However, the evaluation of corrosion inhibition efficiency is not amenable to standardization because in many respects, it is significantly different from corrosion testing. The main reasons are that the addition of an inhibitor to a corroding system requires physical stability to be efficiently dosed, thermal stability, chemical stability for storage, and compatibility with the corroding medium in the industrial process under consideration. Additionally, green inhibitors are generally complex molecules that are affected by the source of plant materials. Therefore, the evaluation of corrosion inhibitors must be specific, not only to a process but also to a given processing plant. All references listed in Table 1 regarding reports on corrosion inhibitors in saline solutions use traditional laboratory testing methods under standard conditions of mixing and constant temperature during variable time periods. The evaluation of inhibition performance using testing methods under harsh conditions (i.e., rotating cage, on-line testing, etc.) is only reported for sweet corrosion cases that apply to oilfield conditions [185,186]. Similarly, for this industry, guidelines for testing corrosion inhibitors have been developed [187]. In the context of water scarcity for urban and industrial applications, the evaluation of the performances of corrosion inhibitors in seawater pipelines is pertinent and highly relevant. In this respect, in Northern Chile, a total 12,000 L/s of seawater and desalinated water are currently being pumped through pipelines [188]. For these conditions, the widespread use of carbon steel, such as API5LX70 or similar, combined with corrosion prevention measures such as lining, corrosion inhibitors/biocides, and anodic protection, is standard practice for transportation seawater and desalination water [189]. Different zinc poly and/or phosphates-based formulations is the preferred choice as corrosion inhibitors [190]. Given the increasing trend of seawater use in urban and industrial areas, there is a need to develop new guidelines for seawater and desalinated water transport. These guidelines should include metallic material selection, methods of corrosion prevention, and restrictions for the use of hazardous chemicals in the water. In this respect the green inhibitors show a promise future.

## 8. Challenges and Future Directions

**Figure 16:**
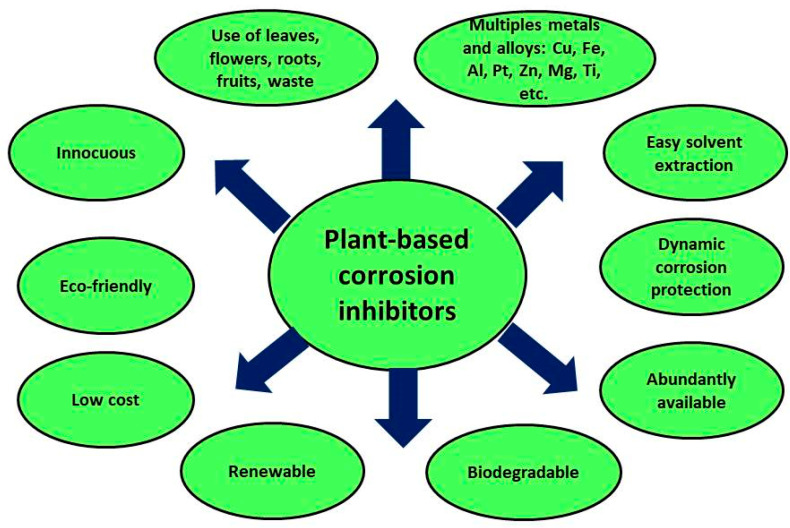
Advantages of using plant extracts as corrosion inhibitors.

The degradation of materials through corrosion caused by electrochemical and/or chemical reactions are triggered when metals come into direct contact with harsh environments, such as seawater. This poses a significant challenge across various industries, particularly in chemistry and metallurgy. This issue can also extend to medical applications where metal biomaterials are in contact with biological environments [191]. In recent years, there has been a push towards exploring sustainable and environmentally friendly solutions, leading to research on green corrosion inhibitors, also known as ecological corrosion inhibitors. These inhibitors, which are derived from natural sources, are environmentally benign and exhibit multiples advantages, as shown in Figure 16, such as high biodegradability, low cost, wide applicability for multiple metal and alloys, and others. However, their effectiveness in saline environments is often limited compared to that of conventional chemical inhibitors because of the weaker adsorption of organic molecules on metal surfaces, resulting in reduced protection against highly corrosive agents.

This limitation stems from the molecular structures of the green inhibitors, which fail to provide robust coverage on the metal surface, leading to the formation of weak protective films. Although these films may be stable in some cases, they often lack stability in corrosion rates over time, raising doubts about their viability as an alternative for corrosion management. Understanding the reaction mechanisms and effects of the intermediate products formed during the use of green inhibitors is crucial, as these intermediates influence their adsorption capacity on metal surfaces. However, studying these mechanisms is challenging because of the complex composition of natural extracts, which contain a mixture of products such as anthocyanins, tannins, saponins, terpenoids, polyphenols, and alkaloids, among others, with selectivity and distribution depending largely on the extraction methods. Furthermore, compatibility studies between green corrosion inhibitors and various types of coatings are essential for the design of effective corrosion protection systems. Some green inhibitors may interact with coatings, compromising their effectiveness and leading to accelerated modification or delamination of protective layers. Although the biodegradability of green inhibitors is desirable, it can also be a limitation in industrial environments requiring long-term inhibitory action.

To address these challenges, ongoing research has explored a wide range of natural products, including plant extracts, biomolecules, and biologically derived polymers, to design inhibitors with improved performance and selectivity, ensuring efficient and uniform coverage on metal surfaces. Advances in quantum chemistry have facilitated the understanding of molecule–metal interactions and the prediction of green inhibitor behavior. Comprehensive financial analyses, from pilot-scale validation to full-scale industrial implementation, are essential for assessing economic viability, with predictive studies using simulation tools that provide valuable insights.

## 9. Conclusions

Green corrosion inhibitors offer immense potential in addressing climate change and reducing the impact on human health owing to their biodegradable nature and environmentally friendly characteristics when combatting metal corrosion in aggressive environments. This review has focused on plant-based extract corrosion inhibitors for metallic materials in aqueous saline media. The analysis and discussion cover topics such as corrosion inhibitor attributes, corrosion mechanisms, inhibition in NaCl solutions, electrochemical and surface characterization techniques, theoretical calculus by DFT, corrosion testing standards, and general guidelines for corrosion inhibitor selection. In this context, extracts of different plant materials, such as roots, flowers, leaves, and seeds have demonstrated inhibition efficiencies close to 90%. Most of these inhibitors act through a mixed effect, limiting both anodic and cathodic reactions, with adsorption characteristics best described by Langmuir, Freundlich and Temkin isotherms. As indicated in the review, emerging testing methods and technologies are relevant in understanding the complex occurrence of corrosion and its protection by using green inhibitors.

Despite significant progress in designing, developing, and testing these inhibitors, several limitations and challenges persist, necessitating prompt resolution. Continued growth in quantum analysis is vital, as it serves as a powerful tool for studying the behavior of green corrosion inhibitors, enhancing our understanding of their interactions with metallic surfaces. By focusing on improving the efficiency, environmental sensitivity, compatibility, temperature and pressure dependencies, biodegradability, and cost-effectiveness of green inhibitors, we can develop sustainable and effective anticorrosion strategies in chloride-rich environments, contributing to a more ecological and sustainable future. Additionally, the continuous increase in seawater use in industries necessitates the development of guidelines for the selection of metallic materials, methods of corrosion prevention, and restrictions regarding the use of inhibitors. Given the advantages of green corrosion inhibitors, plant-based inhibitors show a promise future.

## Figures and Tables

**Table 2:** Electrochemical parameters derived from Tafel plots of the mild steel samples immersed in 3.5% NaCl with and without Persian licorice extract after 24 h immersion [134].

Solution	E_corr_ vs. Ag/AgCl (mV)	i_corr_ (µA/cm^2^)	βa (V/dec)	–βc (V/dec)
blank solution	–718 ± 32.1	18.6 ± 0.4	0.21 ± 0.008	0.43 ± 0.03
200 ppm	–680 ± 22.3	6.7 ± 0.3	0.09 ± 0.006	0.21 ± 0.03
400 ppm	–621 ± 17.8	5.3 ± 0.3	0.10 ± 0.006	0.17 ± 0.04
600 ppm	–590 ± 14.4	4.6 ± 0.2	0.10 ± 0.002	0.15 ± 0.02

## Data Availability

No new data were created or analyzed in this study.
